# Gut microbiota, an emergent target to shape the efficiency of cancer therapy

**DOI:** 10.37349/etat.2023.00132

**Published:** 2023-04-26

**Authors:** Soumaya Kouidhi, Oumaima Zidi, Zeineb Belkhiria, Henda Rais, Aida Ayadi, Farhat Ben Ayed, Amor Mosbah, Ameur Cherif, Amel Ben Ammar El Gaaied

**Affiliations:** 1 Laboratory BVBGR-LR11ES31, Biotechnopole Sidi Thabet, University Manouba, ISBST, Ariana 2020, Tunisia; 2 Association Tunisienne de Lutte contre le Cancer (ATCC), Tunis, Tunisia; 3 Department of Biologu, Faculty of Sciences of Tunis, University of Tunis El Manar, Tunis 1068, Tunisia; 4 Medical Imaging Center, IBN Zohr, City El Khadhra 1003, Tunisia; 5 Service d’Oncologie Médicale, Hôpital Salah-Azaïz, Tunis 1006, Tunisia; 6 Department of Pathology, Abderrahman Mami Hospital, University of Tunis El Manar, Ariana 2080, Tunisia; 7 Laboratory of Genetics, Immunology and Human Pathology, Department of Biology, Faculty of Sciences of Tunis, University of Tunis El Manar, Tunis 1068, Tunisia; Université Paris-Saclay, France

**Keywords:** Cancer, gut microbiota, dysbiosis, chemotherapy, immunotherapy

## Abstract

It is now well-acknowledged that microbiota has a profound influence on both human health and illness. The gut microbiota has recently come to light as a crucial element that influences cancer through a variety of mechanisms. The connections between the microbiome and cancer therapy are further highlighted by a number of preclinical and clinical evidence, suggesting that these complicated interactions may vary by cancer type, treatment, or even by tumor stage. The paradoxical relationship between gut microbiota and cancer therapies is that in some cancers, the gut microbiota may be necessary to maintain therapeutic efficacy, whereas, in other cancers, gut microbiota depletion significantly increases efficacy. Actually, mounting research has shown that the gut microbiota plays a crucial role in regulating the host immune response and boosting the efficacy of anticancer medications like chemotherapy and immunotherapy. Therefore, gut microbiota modulation, which aims to restore gut microbial balance, is a viable technique for cancer prevention and therapy given the expanding understanding of how the gut microbiome regulates treatment response and contributes to carcinogenesis. This review will provide an outline of the gut microbiota’s role in health and disease, along with a summary of the most recent research on how it may influence the effectiveness of various anticancer medicines and affect the growth of cancer. This study will next cover the newly developed microbiota-targeting strategies including prebiotics, probiotics, and fecal microbiota transplantation (FMT) to enhance anticancer therapy effectiveness, given its significance.

## Introduction

Although precision medicine has made enormous strides in the treatment of cancer, and disease-free survival has shown clinical improvements, the required success rate has not yet been reached. Cancer patients still face resistance to therapy that limits reaching optimal cures, with high tumor recurrence [1]. Several mechanisms of resistance caused by chemotherapy and immunotherapy have been intensively described [1]. These side effects were related to genetic and epigenetic alterations or DNA damage repair, modifications, deregulation of cell death, unfavorable immune responses, and complex interactions within the tumor microenvironment (TME) [2, 3]. Interestingly, emergent mechanisms of cancer therapy resistance have been recently proposed. Emergent proof from preclinical and clinical studies has focused on the key role of gut microbiota in anticancer remedy response via modulating drug efficacy, abolishing the anticancer effect, and interceding toxicity [4]. Accumulating evidence has discovered that an altered intestine microbiome is related to resistance to chemotherapy or immune checkpoint inhibitors (ICIs) [5–7]. Therefore, it is not surprising that the latest investigations spotlight the possibility of modulating the gut microbiota to overcome the failure of remedy and poor cases issues, increase the efficacy of cancer treatment, and repair the original healthy microbiota [8–10].

Hence, a deep understanding of the interplay between microbiota and cancer treatment could help to implement new strategies for cancer prevention and patients’ stratification for more efficacy and less complication due to therapy.

In this review, authors first provide the scientific evidence highlighting the critical role of the gut microbiome in cancer establishing the links between gut microorganisms and cancer growth, immune responses, as well as the efficacy of anticancer treatment mainly chemotherapy and immunotherapy. This review will next go over the latest microbiome-targeting strategies, including probiotics and prebiotics, antibiotics, and fecal microbiota transplantation (FMT), which may improve the effectiveness of cancer treatments.

## Human gut microbiota

From birth, many bacteria that are primarily found on the skin, mouth cavity, vagina, and stomach coexist with humans [11]. Nevertheless, the gastrointestinal (GI) tract as well as distal organs including the brain, liver, and pancreas are all impacted by the gut microbiota, which has become an important factor in human health [12, 13]. Since the introduction of culture-independent techniques like omics, we have gained a greater understanding of the structure and function of the human gut microbiota [14–16]. The adult human gut microbiota incorporates 10^13^–10^14^ microorganisms/mL of luminal content, with an estimated biomass of about 1.5 kg [17]. Bacteria predominate microbial communities, as is well known. However, a more recent sharp increase in studies examining the involvement of viral and fungal components of the microbiome suggests that viruses, archaea, and fungus may potentially play crucial roles in preserving gut homeostasis [18]. Though it has been hypothesized that the virome may control the microbiome and affect bacterial complexity and population dynamics. It is likely that the most common bacteria are eliminated by their phages [19]. Thus, phages have a significant impact on the bacterial communities in the intestine [19]. However, because of their reduced abundance and the lack of specialized techniques and curated reference databases for their identification and classification, the viral and fungal biomes have not been well defined up to this point [20].

**Figure 1.**
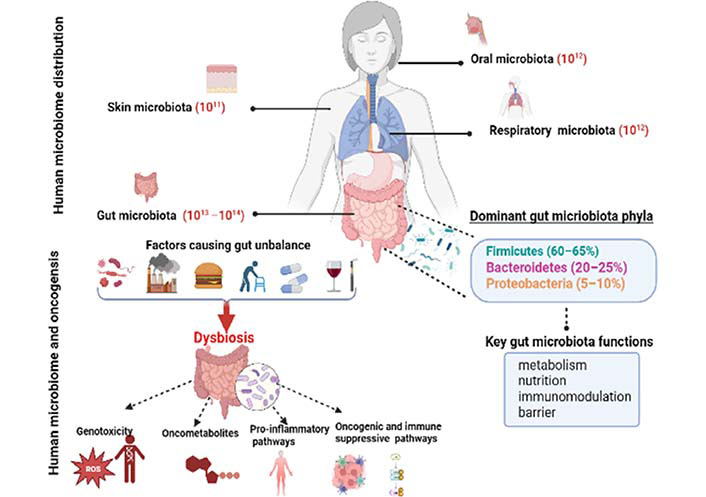
Human gut microbiota composition and its mechanistic roles in cancer

The microbiota’s structure remains constant throughout adulthood despite changes brought on by environmental and developmental factors [21]. Firmicutes (60–65%), Bacteroidetes (20–25%), and Proteobacteria (5–10%) are the phyla with the highest representation [22]. Lately, the microbiota is considered as an essential organ and has been associated with health and disease status. When the intestinal ecosystem is in good microbial equilibrium, we talk about “eubiosis” which is associated with good health. Indeed, it is sometimes considered as our “forgotten organ” [23]. In eubiosis, the gut microbiota provides a range of beneficial properties to the host, and its roles in human health have been recently investigated and described in different aspects. Actually, these microbes have important roles in preserving the mucosal barrier’s integrity, delivering nutrients including vitamins, short-chain fatty acids (SCFAs), and secondary bile acids, or protecting against infections [24–26]. Even more crucially, the gut microbiota interacts with the immune system, sending messages to encourage immune cell maturation and the appropriate development of immunological functions [27]. The composition of the microbiota is susceptible to host and environmental selective factors such as alteration in the immune system, host mutations, diet, antibiotic therapy, hospitalization, and chemical exposure. Such factors lead to impaired microbiota and then we talk about dysbiosis. A shift in the gut microbiota’s composition and functions, known as dysbiosis, is defined by a decline in the proportion of commensals and symbionts and/or an increase in the proportion of pathobionts [28]. It has long been known that there is a connection between microbiota and both health and disease. In fact, as Hippocrates, the father of medicine, once said, “all disease begins in the gut”. Recently, the literature has linked dysbiosis to the development of several diseases such as obesity and metabolic disorders [29], type 2 diabetes [30], inflammatory bowel diseases [31], and cancers [32]. Zitvogel et al. [33] showed that the gut microbiota can influence oncogenesis through a variety of mechanisms, including the direct oncogenic effects of microorganisms and their products, the alteration of circulating pro-carcinogen metabolites, the stimulation of the host’s production of trophic factors, and finally, the stimulation of immune-suppressive and pro-inflammatory pathways disrupts the host’s ability to detect malignancy (Figure 1).

## How gut microbiota plays a key role in cancer development?

### What biological processes are deregulated in cancers?

Cancers are diseases that can affect different organs and are due to abnormal behaviour of so-called transformed cells that have acquired this behaviour as a result of different somatic alterations of a genetic and epigenetic nature [34, 35]. Thus, starting from a normal state, a cell can undergo the action of carcinogenic agents that cause genetic abnormalities affecting the sequence of DNA and the expression of genes [36, 37]. Following this stage that generates initiated cells, the carcinogenesis occurs on this initiated already cells which will develop into a tumor under the action of the microenvironment which ensures the promotion of cancer by providing the initiated cell with proliferation signals that will perpetuate the anomalies during divisions, and thus ensure the progeny of the abnormal cell [38–40]. This progeny will accumulate in its turn new genetic abnormalities. Carcinogenesis is a dynamic process that, at each stage, selects a new cell that has undergone one or more alterations [40]. Each cancer originates from the alteration of 10 to 20 genes [41]. These alterations occur successively, each of them favoring the next. This sequence of alterations usually occurs over a very long chronological range. Deregulated genes in cancers affect signaling pathways and molecular and metabolic mechanisms leading to loss of control of cell divisions, immortality, resistance to apoptosis, and metabolic reprogramming [42–44].

The cancer progresses by giving clinical consequences: it grows within precise histological limits (we speak of cancer *in situ*), then exceeds them and becomes invasive. The increase in tumor size is accompanied by hypoxia which stimulates the expression of pro-angiogenic factors and inhibits the expression or production of anti-angiogenic factors, thanks to the stabilization of hypoxia-inducible factor 1-alpha (HIF1α). Tumor angiogenesis refers to the ability of tumor cells to stimulate the appearance and development of blood vessels and thus promote their own growth [45, 46]. Tumor vascularization facilitates metastases which are secondary tumor formations that derive from cancer cells that have acquired the property of disassociating [epithelial mesenchymal transition (EMT) transition] from the primary tumor and have implanted remotely through a cellular mechanism called EMT [47, 48].

Hanahan and Weinberg [49] defined the characteristics of cancer cells and tumors which share common hallmarks: self-sufficiency in growth signals, insensitivity to growth inhibitor signals, ability to avoid apoptosis, capacity for indefinite replication, induction of angiogenesis, ability to metastasize, escape from the immune system, and deregulation of cellular energy metabolism. All these characteristics are sustained by tumor cell genome instability and tumor interaction with its micro-environment particularly inflammation and nutrient and energetic sources.

### What epidemiological and clinical arguments for the involvement of the microbiota in cancers?

#### Dysbiosis of gut microbiota and colorectal cancer

The most studied examples concern the intestinal microbiota in relation to the occurrence of digestive cancers and particularly colorectal cancer (CRC). In germ-free rats that developed fewer chemically induced colorectal tumors than conventional rats, Weisburger et al. [50] presented the first study associating the gut microbiota with CRC. These findings have been verified in animals prone to CRC [51]. In humans, such a link between gut microbiota and CRC has been reported in several studies (Table 1). In a healthy gut microbiota, the dominant bacteria phyla include Firmucutes, Proteobacteria, Bacteroidetes, and Actinobacteria, with a diverse structure at the genus and species levels in gut micro-communities. To explore the role of gut microbiota in the etiology of cancer, researchers utilize metagenomic analysis that allows comparison of microbiota composition between healthy populations and those with cancer diseases, investigating cancer tissues along with matched healthy tissues.

**Table 1.** Involvement of gut microbiota dysbiosis in CRC

**Phyla and species concerned by dysbiosis in CRC**	**Reference**
Infection with *S. bovis* a risk factor for colon tumors	[52]
High enrichment of Fusobacteria in colorectal carcinoma tissue	[53]
ETBF, and *Fusobacterium nucletum* are highly expressed in CRC tissue	[54]
Firmicutes and Fusobacteria were over-represented whereas Proteobacteria was under-represented in CRC patients; *Lactococcus* and *Fusobacterium* is more abundant while *Pseudomonas* and *Escherichia-Shigella* reduced in cancerous tissues *versus* adjacent tissues	[55]
Mucosa-associated *E. coli* of the B2 phylogroup more prevalent in CRC tissues	[56]
*Fusobacterium nucleatum* isolated from tumor tissue and proved to be invasive in the *in vitro* experiments	[57]
*B. fragilis*, *Enterococcus Escherichia-Shigella*, *Klebsiella*, *Streptococcus*, and *Peptostreptococcus* is abundant in CRC patients, while *Roseburia-* and *Lachnospiraceae-*related OTUs more abundant in healthy controls	[58]
CRC patients have a lower microbiota diversity and *Clostridia* abundance, with a high abundance of *Fusobacterium* and *Porphyromonas* at the genus level	[59]
*Bifidobacterium*, *Faecalibacterium*, and *Blautia* reduction, while *Fusobacterium* enrichment in the CRC patients; stool samples in CRC patients enriched with *Paraprevotella*, *Eubacterium*	[60]
Association of *Clostridioides difficile* with CRC revealed by anti-tcdB antibodies in plasma, particularly the IgA level; anti-tcdB antibodies as candidate serologic markers for CRC	[61]
Bacteroidetes cluster 1 and Firmicutes cluster 1 decreased in CRC mucosa, whereas Bacteroidetes cluster 2, Firmicutes cluster 2, pathogen cluster, and *Prevote*lla cluster increased in CRC mucosa; compositional alterations in the microbiota differ between distal and proximal cancers	[62]

*S. bovis*: *Streptococcus bovis*; ETBF: enterotoxigenic *Bacteroides fragilis*; *E. coli*: *Escherichia coli*; *F. nucleatum*: *Fusobacterium nucleatum*; *B. fragilis*: *Bacteroides fragilis*; OTUs: operational taxonomic units; tcdB: clostridium difficile toxin B; IgA: immunoglobulin A

Contrary to gastric carcinogenesis, which seems to result from a single pathogen [*Helicobacter pylori* (*H. pylori*)] [63], the composition of the gut microbiota and more particularly the imbalance between groups of species rather than a particular species seem to be decisive in defining dysbiosis that can generate CRC cancer.

There is little agreement regarding the changes seen in this cancer, despite the fact that many studies have documented dysbiosis in patients with CRC. But several species, like *S. bovis*, *H. pylori*, *B. fragilis*, *Enterococcus faecalis* (*E. faecalis*), *Clostridium septicum* (*C. septicum*), *Fusobacterium* spp., and *E. coli* [64], and more recently *Clostridioides difficile*, have been specifically considered to have a role in CRC [61].

Overall, gut microbiota diversity decrease seems to be associated with CRC. In contrast to *F. nucleatum*, which has been linked to disease, *Bacteroidetes fragilis* acts as a protective factor in the gut by regulating inflammatory immune responses. A major question asked was whether dysbiosis is a risk factor for cancer or whether the tumor is a microenvironment that influences the composition of the microbiota. In fact, it is not to be excluded that in the context of a crosstalk, a dysbiosis makes the bed of cancer which in turn accentuates this imbalance of the microbiota that can participate in the progression of the tumor. To investigate this point, it is interesting to know if dysbiosis is associated with the presentation of cancer.

#### Gut microbiota dysbiosis and CRC clinical presentation

Investigations into the association of gut microbiota dysbiosis with CRC were carried out considering mostly the stage, the grade of the disease, the CpG methylation, and instability of microsatellite profiles, these latter being associated with colorectal carcinogenesis pathway. The case for differences in gut microbiota composition by stage of CRC has been provided by several researchers. It seems that there are microbial metacommunities that change according to the progression of the cancer. According to one study, it is possible to anticipate the shape and function of certain mucosal microbial communities as colorectal neoplasms develop along the adenoma-carcinoma sequence [65]. However, two man species have been particularly associated with the presentation of CRC. Some researches suggest that *E. coli* may contribute to the pathophysiology of CRC. A connection between the tumor-node-metastasis stage and *E. coli* colonization of the mucosa was specifically noted. In addition, it has been shown that pathogenic cyclomodulin-positive *E. coli* bacteria were more common in the mucosa of individuals with stage III/IV colon cancer than in stage I colon cancer patients. The correlation between the tumor proliferative index and *E. coli* colonization of the mucosa was also significant [66].

Another study that demonstrated that advanced colorectal neoplasia patients’ large intestine mucosa is colonized with more virulent strains of *E. coli* and that higher bacteriocin production was associated with an increasing stage of CRC [assessed according to tumor, nodes and metastases (TNM) classification], supported these findings [67]. Other species were also involved in the progression and aggressiveness of CRC. *Fusobacterium* and also ETBF showed important associations with CRC clinicopathological features. *Fusobacterium* has been particularly involved in such features. Increased levels of *F. nucleatum* were shown to be related to CRC progression and to CRC patient survival [68]. *Fusobacterium* spp. levels were significantly greater in late stage (III/IV) colorectal malignancies, and an excess of *F. nucleatum* has been linked to a higher risk of lymph node metastases in colon cancer [68]. Regardless of the histology, *F. nucleatum* was discovered in premalignant colorectal lesions, however, it was more frequently found in lesions with high CpG island methylator phenotype (CIMP) levels. Additionally, *F. nucleatum* increased with histological grade, indicating that it might aid in the development of colorectal neoplasia. The hypothesis of the “colorectal continuum” may be supported by the presence of *F. nucleatum* positive in CR tumors [69]. Furthermore, mounting data suggests that *F. nucleatum* may contribute to particular molecular subgroups of CRCs, including the CIMP and microsatellite instability (MSI) [70].

#### Gut microbiota dysbiosis and other cancers

The pathogenesis of colon, gastric, esophageal, pancreatic, laryngeal, breast, lung, and gallbladder carcinomas has been linked to gut microbial dysbiosis. This change appears to be intimately related to the host inflammation brought on by microbial dysbiosis. We will present some here the cases of lung and breast cancers (BCs) which are the most frequent around the world.

In contrast to the control group, lung cancer patients had a much lower relative abundance of several gut microorganisms. These results imply that there may be some indirect links between lung cancer and gut microbiome [71]. Lung and intestinal flora have an impact on how lung cancer develops, is treated, and is prognosed. These factors will help develop new lung cancer prevention, detection, and treatment methods [72].

BC and gut microbial dysbiosis have been linked in several studies. As compared to healthy women, the microbiota of BC patients is different, suggesting that specific bacteria may contribute to the development of the disease. Elevated serum levels of estrogen can be modified by the gut flora. Contrarily, chemicals that resemble estrogen may encourage the growth of specific bacterial species. As a result, there may be a synergistic effect between the microbiota and both endogenous hormones and estrogen-like substances that raise the chance of developing hormone-related disorders like BC [73]. According to other research, breast tissue has a unique microbiome, with some species being elevated in the breast tissue itself as well as the gut bacteria of BC patients. More significantly, the microbiome of the breast and its associated tissues may act as potential indicators for detecting and staging BC [74]. It has been hypothesized that BC development may be influenced by changes in the mammary and intestinal microbiota. Bacteria from the phyla Proteobacteria, Firmicutes, Actinobacteria, and *Lactococcus* spp. are abundant in healthy breast tissue and may therefore have potential preventive effects against BC. But in malignant mammary tissues, some bacteria are more prevalent. The dysbiosis of the gut microbiota, on the other hand, has been associated with BC because some gut bacteria can affect the synthesis of advantageous metabolites and interfere with the metabolism of estrogen in the stomach. Such surprising connections between BC and gut microbiota in the breast and BC suggest potential solutions for managing BC, such as through the use of probiotics, both in terms of prevention and management [75]. It has been established that the compositional abundance of some bacterial families and the synthesis of microbial metabolites vary in BC. It might be said that microbial dysbiosis appears to be linked to the development of BC [76].

### By which biological mechanisms does the gut microbiota interfere with the occurrence and progression of cancers?

How bacterial microbiota modifications could represent novel approaches for risk assessment and, may serve as prognosis markers and/or targets for innovative therapeutic strategies in cancer? To this aim, we should better understand the pro-carcinogenic effects of gut bacteria involved in microbiota dysbiosis. Through a number of ways, the bacterial microbiota causes colorectal carcinogenesis. In this review, we explore the connections between the bacterial microbiota and colorectal carcinogenesis, concentrating on dysbiosis and the potentially cancer-causing traits of bacteria, such as genotoxicity and other virulence factors, and oxidative stress. On the other hand, gut microbiota dysbiosis may be involved in tumor promotion and progression by influencing the tumor micro-environment through inflammation, host immune defenses modulation, and bacterial-derived metabolism [64].

According to such potential effects, two main hypotheses have emerged to explain the impact of dysbiosis on CRC initiation and progression. One hypothesis holds that a dysbiotic microbial population can cause cancer by altering the microbiome as a whole, triggering inflammatory reactions, and transforming epithelial cells. The “driver-passenger” paradigm proposes that intestine “bacteria drivers”, initiate CRC by inducing epithelial DNA damage and epigenetic alterations leading to tumorigenesis. The tumor generates a microenvironment that in its turn promotes the proliferation of “passenger bacteria” that takes advantage of such tumor [77].

#### Microbiota dysbiosis and genotoxicity

Instability in the genome and the proliferation of epithelial cells are two factors in the development of CRC that are brought on by these mechanisms, which also have a variety of cellular effects and affect the host’s defenses. The dysbiotic bacteria implicated in cancer initiation produce toxic molecules that can induce DNA damage leading to transformation. Cyclomodulins are bacteria toxins that can induce DNA damage, interfere with the cell cycle, and/or apoptosis. Among such toxins, cytolethal distending toxin (CDT) and colibactin can directly damage DNA and induce genomic instability. Considered true genotoxins they are able to provoke double-strand DNA breaks [78, 79].

Oxidative stress could be another mechanism involved in carcinogenesis. Highly reactive molecules called reactive oxygen species (ROS) are created from oxygen. According to research, ROS induction plays a crucial part in CRC that is connected to the microbiome. Because hydroxyl radicals are potent mutagens that may cause DNA breaks, point mutations, and protein-DNA crosslinking, enterococci species, particularly *E. faecalis*, can produce them [80], which can contribute to genomic instability in CRC [81, 82]. The gut microbiota also promotes host-derived production of nitric oxide and its secondary nitric oxide synthases (NOS), which can induce DNA damage. Some bacterial species such as *Lactobacilli* and *Bifidobacteria* can directly generate NOS [83]. Some enteropathogenic *E. coli* strains have the potential to downregulate the DNA measles, mumps, and rubella (MMR) system, demonstrating the connection between oxidative stress and DNA repair mechanisms [84]. The accumulation of mutations linked to the development of CRC can result from this downregulation of the MMR repair mechanism [85]. Viljoen et al. [54] also noted that *F. nucleatum* colonization was more prevalent in the colon of CRC patients who had a phenotype associated with MSI caused by MMR deficiency.

These findings support the theory that the gut bacteria and DNA repair mechanism interact to cause CRC.

#### Interference of gut microbiota with signaling pathways

The dysbiotic bacteria implicated in cancer produce toxic molecules that interfere with proliferation and apoptosis signaling pathways leading to cell transformation. Some bacteria toxins may be involved in colorectal carcinogenesis through the activation of carcinogenesis-promoting pathways. The examples of cytotoxin-associated gene A (*CagA*) or vacuolating cytotoxin gene A (*VacA*) produced by some *H. pylori* strains are well known [86]. Through direct binding, phosphorylation of signaling proteins, and methylation of tumor suppressor genes, *CagA* impacts the expression or function of critical proteins in oncogenic or tumor suppressor signaling pathways [87, 88]. *VacA* activates the signaling pathway of the mitogen-activated protein kinases (MAPK) p38, and extracellular signal-related kinases 1 and 2 (ERK1/2). Moreover, *VacA* activates a signaling pathway involving G-protein-coupled receptor kinase-interactor-1 (Git1) leading to the upregulation of the β-catenin signaling pathway involved in CRC [89].

The *B. fragilis* toxin (BFT) is one more illustration of a bacterial toxin that contributes to the development of CRC. In order to activate the Wnt and nuclear factor-kappa B (NF-κB) pathways, the zinc-dependent metalloprotease toxin BFT must attach to a particular colonic epithelial receptor. These outcomes result in accelerated cell division, epithelial release of pro-inflammatory mediators, and development of DNA damage [90, 91].

#### Microbiota as tumor micro environment

As widely demonstrated, the microenvironment plays a determining role in the promotion of tumors and the progression of cancer. According to the theory of the carcinogenic effect of the dysbiotic microbial community, dysbiosis could be sufficient to promote cancer, suggesting that the microbial environment of the tumor should play an important role in its progression.

Among the described mechanisms, it is necessary to consider inflammation and host immune defenses modulation, as key factors in the interactions between the gut microbiota and CRC. It is now widely accepted that people with inflammatory bowel illness have a higher chance of getting CRC. Since their microbiota underwent significant alterations throughout the development of CRC, inflammation may be a result of the host’s reaction to those changes [92, 93]. One of the microorganisms most closely linked to inflammatory bowel illness is *E. coli*. In individuals with inflammatory bowel illness, it has been seen that adherent and invasive *E. coli* abnormally colonize the gut mucosa. The bacterial control of inflammation in colorectal carcinogenesis is supported by the ability of CRC-associated *E. coli* to promote the expression of the pro-inflammatory gene cyclooxygenase-2 (*COX-2*) in macrophages [94, 95]. The gut microbiota participates in metabolic processes, and it is acknowledged that CRC development is significantly influenced by microbial-derived metabolism [96]. By controlling the production of secondary bile acids, the metabolic activation of carcinogenic substances, dietary phytochemicals, and xenobiotics, hormone metabolism, and the regulation of inflammatory pathways, these metabolic activities may influence colorectal carcinogenesis [97].

Intestinal epithelial cell differentiation and proliferation, energy storage from food sources, vitamin synthesis, ion absorption, and mucus formation are all potential outcomes of these processes [98].

### Tumoral microbiome, a new constituent of the TME

The TME is constituted of all the non-tumor cells and soluble molecules surrounding the tumor. It’s classically known to include pericytes, fibroblasts, immune cells, adipocytes, vascular and lymphatic endothelial cells, and factors secreted by both tumor and non-tumor cells [99, 100]. Recently, researchers reported that the microbiome is a newly recognized component of the TME [101]. Despite the fact that the intestinal microbiome has been established as an essential biomarker of cancer development and therapeutic response, less is known about the role of the microbiome in the tumor environment [101]. Emerging evidences had reported that the local microbiota constitutes an important part of the TME across many types of cancer including laryngeal, esophageal, gastric, and CRCs, as well as primary liver cancer [102]. In order to really understand its impact on cancer, it is important to understand how the microbiome can shape the TME and interact with cancer cells.

In a study conducted by Wei et al. [103], the TME was reported to ease the growth of anaerobic and facultative anaerobic bacteria such as *Clostridia*, while necrotic areas of the tumor were thought to cause chemotactic compounds releasing attracting then bacterial invasion [104]. The leaky vasculature of tumoral tissues also permits bacteria to penetrate and the lack of immune cells may allow their growth [105]. Distantly, the gut microbiota was demonstrated to modulate the fate of tumors in distant organs by producing metabolites and immune signals that enter the circulation to reach tumors and become a part of their TME [106, 107]. These microbial metabolites may regulate carcinogenesis by interacting with other components of the TME and participating in the immune response or angiogenesis [108–110].

#### TME in CRC

Mucosal tumors are in direct contact with bacteria and therefore are susceptible to influence from the microbiome. In the gut, CRC is exposed to various bacterial species that’s why it has been extensively studied. Interestingly, the tumor tissue microbiome is not fully consistent with the composition of the fecal microbiota [62]. In CRC tissues, Fusobacteria and Firmicutes are increased, while the abundance of taxa including Fusobacteria, *Providencia*, and Actinobacteria are altered between cancerous and adjacent normal tissue [111, 55]. In numerous studies, the presence of these bacteria in colonic tumor tissues is associated with histologic grade, T cell infiltration, resistance to chemotherapy, and poorer overall survival [112–114]. In colonic tumors, *F. nucleatum* has been reported to be enriched and it is likely to have translocated from feces in the gut into colonic epithelium, inducing proinflammatory and oncogenic pathways [115, 116]. It expresses an adhesion protein called FadA, which can bond to E-cadherin and permit the bacteria to invade CRC cells, causing β-catenin regulated transcription of the oncogenes myelocytomatosis oncogene (Myc) and Cyclin D1, and increased cancer cell proliferation [115, 116]. In mouse models, *F. nucleatum* has been shown to induce colorectal tumor via the microRNA (miRNA)—miR21, inhibitors of which prevented CRC cell line proliferation and invasion [116]. miRNAs have been shown different expressions between normal and tumoral colonic tissues, and have been correlated with the microbiome profile of the colon, providing another mechanism by which the gut epithelium can interact with bacteria [115].

#### TME in non-mucosal tissues

Gut microbiota dysbiosis has been long related to decreases in butyrate production within several cancer types, therefore, decreases in butyrate may cause a leakiness of the intestinal epithelium which allows bacteria to migrate to distant tissues via the vasculature [117]. Bacteria have been found in distant tumoral tissues of the intestine such as biliary, esophageal, breast, and cervical cancer, although their significance remains unclear [118, 119]. A recent study analyzing seven human tumor types disclosed a specific microbiome composition in each, and that most bacteria were localized intracellularly within cancer and immune cells of the TME [120]. These findings point out a strong physical relationship between microorganisms and cancer cells at a local level. In agreement with this, human CRC shows the same microbiome composition with its metastatic lesions in the liver, suggesting that the microbiome is able to travel with the primary tumor cells to distant sites [120]. Recently, intratumoral CRC-associated *E. coli* was shown to enter the liver after gut vascular barrier disruption. Once in the liver, *E. coli* were reported to prime the liver microenvironment through the recruitment of innate and inflammatory immune cells to directly promote metastasis [121]. Furthermore, oncogenic bacteria ascendant from the cervix were reported able to reach and colonize the uterus and ovaries during carcinogenesis [122]. In a recent study, *Klebsiella pneumoniae* and some common denominators (*Enterobacter cloacae*, *Citrobacter freundii*, and *Fusobacterium*) were linked with pancreatic cancer as well as BC [120]. These cancer-associated microbes were found to reach the pancreas via peri-intestinal translocation through the pancreatic duct due to gut epithelial barrier damage [123]. Moreover, long-term pancreatic ductal adenocarcinoma survivors had higher intratumoral bacterial diversity and the microbiome signature was significantly different from that of short-term survivors [13]. Interestingly, three enriched genera were identified in long-term survivors (*Saccharopolyspora*, *Pseudoxanthomonas*, and *Streptomyces*), which had a positive correlation with the number of CD8^+^ T cells, suggesting their role in the antitumoral immune response [13]. Functionally, the connections between gut microbiota and intratumoral microbes of the TME are only beginning to be explored and merit further mechanistic investigation.

### Gut virome and cancer

The human virome includes both eukaryotic and prokaryotic viruses, encompassing both enteric viruses and bacteriophage, yet, there are other types of viruses in the human virome such as archaeal viruses and virophages, which are not as deeply studied due to limited understanding of their function within the environment of the human body [124]. Viruses have been reported to hold strong direct or indirect influence over host health and disease by either directly affecting host cell behavior and structure or by altering bacterial communities [125]. Multiple studies demonstrated the implication of various eukaryotic viruses infectious to humans in carcinogenesis inside and outside the intestinal tract, principally those of the families Papillomaviridae and Herpesviridae as well as hepatitis viruses [126–128]. Infections with these viruses are often one of many contributing factors to carcinogenesis and need many years of persistent infection to develop virus-associated cancers [129]. The gut virome contributes to carcinogenesis by diverse mechanisms that vary widely between viral species. Overall, viral infections promote carcinogenesis by the following mechanisms: insertional mutation in the host genome, induction of inflammation, and modulation of signaling pathways in the infected cells by viral oncogenes [130, 131].

#### Human papillomaviruses carcinogenesis

Papillomaviridae is a family of non-enveloped viruses with double-stranded DNA genomes [132]. The human infection member human papillomaviruses (HPV) has been associated with several types of cancer, most notably cervical cancer [127]. However, only a fraction of the approximately 150 HPV types, the so-called high-risk types such as HPV-16 and -18, are commonly associated with cancer [133]. The main mechanisms for the contribution of high-risk HPV to cervical cancer are the integration of viral DNA into the host genome and the expression of viral oncogenes. For example, the early oncogenes *E6* and *E7* have been shown to degrade the tumor suppressor gene *p53* and the retinoblastoma protein (pRb), inter alia, causing cells to arrest in S phase, leading to genomic instability, aneuploidy, and DNA damage, and thus carcinogenesis [134]. Similar mechanisms are thought to operate in other HPV-associated cancers such as CRC [135]. In addition, *E7* has been reported to induce angiogenesis [136], and *E6* and *E7* also deregulate cellular energy. *E7* leads to the Warburg effect, a shift from standard oxidative phosphorylation-based metabolism to aerobic fermentation [137], and *E6* proteins prolong activation of mammalian target of rapamycin complex 1 (mTORC1) signaling by inhibiting receptor protein tyrosine kinase signaling, making cells “blissfully ignorant” regarding their energy state [138].

#### Herpesviridae carcinogenesis

Viruses of the Herpesviridae family are enveloped in double standard DNA (dsDNA). Various members such as cytomegalovirus (CMV), Epstein-Barr virus (EBV), herpes simplex virus 1/2 (HSV1/2) and HSV6 and HSV7 have been implicated in cancer formation [128, 139, 140]. The strongest association with cancer has been reported for EBV, which has been linked to several cancer types including colorectal [141], esophageal [142], and gastric cancer [142]. EBV is an oncogenic γ-1 herpesvirus associated with a variety of lymphoid malignancies, including a variety of B cell, T cell, and natural killer cell (NK cell) lymphomas and epithelial carcinomas [143]. EBV mimics B cell proliferation and survival signals, enabling it to replicate its genome while remaining latent and immunosilent in host B cells, creating a lifelong persistence that favors its spread [143]. When EBV first infects naive B cells, it adopts a growth program or Late III (Lat III) pattern, and EBV expresses Epstein-Barr nuclear antigen 1 (EBNA1)–EBNA6 as well as latent membrane protein 1 (LMP1), LMP2A, and LMP2B. This expression pattern forces infected cells to become proliferating B cells, allowing EBV episomes to replicate [143].

#### Gut virome and CRC

Recently, more and more evidences have shown that GI cancer can lead to the occurrence and development of cancer, especially GI cancer. Metagenomic analysis of stool samples from patients with CRC revealed a characteristic fecal fibroma, indicating increased abundance and diversity of intestinal fibroids [144]. Enteroviral markers that distinguish CRC from non-CRC controls include orthobuniaviruses, tunalikeviruses, phikzlikeviruses, and other viral genera [144]. Phage enrichment has been demonstrated for inoviruses and tunalikeviruses [144], reported, certain species of inoviruses can regulate the production of bacterial biofilms that contribute to colon carcinogenesis [145] and significantly reduced Enterobacteriaceae phages and crAssphage compared to healthy controls [146]. With regard to eukaryotic viruses, especially human oncogenic viruses, EBV, HPV, human polyomavirus, and CMV showed higher prevalence in CRC tissues compared with non-CRC tissues [147]. Preliminary evidences also suggest that EBV infection may contribute to the development of CRC by inducing mutations in enterocytes [148]. It has been reported that EBV-infected B lymphocytes produce microvesicles containing EBV-derived molecules that translocate to intestinal epithelial cells and subsequently initiate oncogenic transformation [149]. Polyomaviruses have been reported to produce T antigens and inactivate important regulatory tumor suppressor proteins, thereby inducing chromosomal instability and malignant transformation of colonocytes [149].

## Impact of gut microbiota on anticancer therapies

**Figure 2.**
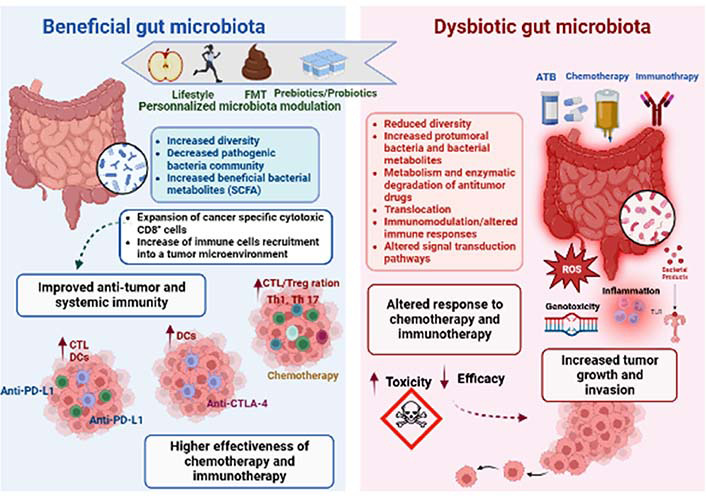
Gut microbiota impacts both anti-cancer chemotherapy and immunotherapy treatments. The gut microbiota composition and function seem to be closely related to patient responsiveness to anti-cancer therapy. The microbial composition is linked to lifestyle and could be altered by several factors, such as antibiotics (ATB), chemotherapy, or immunotherapy treatments. Thus, dysbiosis can be defined as an imbalance in number and types of microbial communities, contributing to tumor development and growth in human beings. Dysbiosis might influence the course of carcinogenesis because microbial activities appear to have an impact on genetic and epigenetic changes that result in dysplasia, clonal expansion, and malignant transformation. The dysbiotic microbiota promotes carcinogenesis in a variety of methods, including by directly activating bacterial toxins like ROS or by indirectly activating inflammatory pathways [via toll like receptor (TLR) and other pattern recognition receptors (PPR)]. Dysbiosis tends to disturb the TME leading to targeting personalized modulation of the microbiota by prebiotics/probiotics, FMT, or lifestyle (diet and exercise) intervention, which could therefore be a potential clinical strategy to enhance the anti-cancer therapeutic response. In fact, it has been proven that a favourable microbiota has the ability to enhance the efficacy of therapeutic treatments, by activating the host anti-tumor immune response. Overall, beneficial bacteria might be key determinants of improving anti-programmed cell death protein-1 (PD-1)/PD-1-ligand 1 (PD-L1), anti-cytotoxic T lymphocyte-associated antigen-4 (CTLA-4), and chemotherapy efficacy. CTL: CD8^+^ T cell (use only in Figure 2); Treg: regulatory T cell; Th1: T-helper 1; DCs: dendritic cells

Apart from the role in carcinogenesis, recent clinical studies focusing on several types of cancer strongly suggest that gut bacteria play a key role in mediating the effects and outcomes of the host response to anti-tumor drugs, especially chemotherapy and immunotherapy [150]. Microbiota modulates therapeutic effectiveness, eliminates the anticancer effect, or mediates drug toxicity, all of which have a significant impact on how cells react to anticancer therapy [6]. Concepts on how microorganisms and anticancer medications interact have undergone paradigm adjustments. These studies have shed light on how many systemic chemotherapies and immunotherapies work, and they have raised hopes that the microbiota may be altered to increase the effectiveness of therapy and lessen adverse effects (Figure 2).

### Role of gut microbiota in chemotherapy response

Despite the scientific progress, we are still lacking literature on the role of the gut microbiota as an anti-cancer therapy. Little is known about microbiota-host-chemotherapy interactions in cancer. According to studies, the gut microbiota can influence how the body reacts to chemotherapy through a variety of processes, such as immunological interactions, xenometabolism, and altered community structure [4]. A study conducted by Alexander et al. [4] has demonstrated that the gut microbiota exerts, through a large suite of chemical signaling cascades, both direct and indirect effects on chemotherapy efficacy and toxicity. Although the mechanisms are not well understood, and in order to explain how bacteria affect chemotherapy treatment effectiveness in terms of translocation, immunomodulation, metabolism, enzymatic degradation, decreased diversity, and ecological variation, Alexander et al. [4] have developed a “TIMER” mechanistic framework [77].

#### Bacterial translocation and immunomodulation to enhance chemotherapy efficacy

The mechanism by which commensal or pathogenic bacteria transcend the gut barrier into the tumoral milieu, where they might increase the side effects of chemotherapy [151], was first documented in the 1960s [152]. In addition, the microbiota can modulate the chemotherapeutic response by modifying the immune response. In fact, it has been demonstrated that the continual interaction of the innate and adaptive immune systems with the gut microbiota at the mucosal surface controls inflammation and sets the immunological tone [153]. However, it has been shown that chemotherapy medications harm the mucosal epithelium, which results in bacterial translocation. This might cause a systemic infection, increasing the host’s exposure to various pathogens and priming the adaptive immune system to respond more favorably to chemotherapy [81, 82]. Immunomodulation can occur by several mechanisms. The most studied mechanisms are bacterial translocation and TH17 cell activation that enhances the action of cyclophosphamide (CTX); the activation of intraluminal myeloid cells that increases oxaliplatin action and microbiota-induced T cell activation can facilitate novel anticancer immunotherapy [154].

Numerous immunological mechanisms have been implicated in the anti-neoplastic actions of the widely used alkylating drug CTX [82, 84]. Using a mouse sarcoma model, research by Viaud et al. [155] revealed that both doxorubicin and CTX shorten intestinal villi and disrupt the intestinal barrier, causing commensal bacteria to translocate into secondary lymphoid organs. They have demonstrated that CTX can change the microbiota of the small intestine, causing bacterial species from the Firmicutes phylum, such as *Roseburia*, *Coprococcus*, and unclassified Lachnospiraaceae, as well as *Lactobacilli* and *Enterococci*, to become less prevalent [156]. Additionally, recent studies reported that a corrupted microbial barrier was more permeable to Gram-positive bacteria leading to the translocation of *Lactobacillus johnsonii*, *Lactobacillus murinus*, and *Enterococcus hirae* (*E. hirae*) from the gut into the lymphoid organs [157]. Translocated bacteria promote TH17 cell development once they reach the spleen and mesenteric lymph nodes, leading to an antitumor adaptive immune response. Recent studies have shown that vancomycin-pretreated mice had less Th17 cells in their spleens than untreated animals, and they have indicated that adoptively transferring pathogenic Th17 cells to the treated mice may restore a therapeutic response [158]. Additionally, a recent clinical study has shown that a memory Th1 immune response to *E. hirae* among patients with end-stage lung and ovarian cancer was proven to be a favorable predictor of progression-free survival [82, 85, 159].

Furthermore, Iida et al. [160] have investigated the impact of microbiota and myeloid cell interaction on chemotherapeutic efficacy in a murine lymphoma model. They have demonstrated that pre-treatment of mice with subcutaneous EL4 lymphoma with antibiotics could reduce both DNA damage caused by oxaliplatin and the expression of genes responsible for ROS generation by myeloid cells [161]. It leads to the conclusion that a healthy microbiota may boost the antitumor impact of oxaliplatin by prepping myeloid cells for ROS release, improving inflammatory cytokine production, and ultimately eradicating tumors [161].

#### Role of bacterial enzymatic degradation and metabolism in anticancer toxicity

The gut microbiota seems to have the potential to directly metabolize chemotherapeutic drugs and indirectly alter host-chemotherapeutic metabolism by modifying the host metabolic milieu [86, 88]. The gut microbiota seems to have the potential to directly metabolism chemotherapeutic drugs and indirectly alter host-chemotherapeutic metabolism by modifying the host metabolic milieu [86, 88]. Recent studies show that changes in the gut microbiota may be the cause of the varying clinical response to fluoropyrimidines, using the *Caenorhabditis elegans* (*C. elegans*) nematode worm as a model for symbiotic host-microbial interactions [161]. García-González et al. [162] tested the impact of either *E. coli* or *Comamonas* bacteria administration in the efficacy of camptothecin (CPT), 5-fluorouracil (5-FU), and 5-fluoro-2’-deoxyuridine (FUDR) in *C. elegans*. The gut microbiota seems to have the potential to directly metabolism chemotherapeutic drugs and indirectly alter host-chemotherapeutic metabolism by modifying the host metabolic milieu [86, 88]. It was found that the response to chemotherapeutic was affected by bacterial species differently. In fact, *C. elegans* fed by *E. coli* was more sensitive to the sterilizing effect of FUDR [161].

Further investigations suggested that microbial metabolism of anticancer drugs could cause severe side effects such as diarrhea, pain, and weight loss leading to dose limitation, which reduces the efficacy of anticancer treatment. Recent studies have demonstrated that irinotecan induced diarrhea in up to 30% of patients requiring dose reduction and, in many cases, the immediate termination of the drug [162]. Irinotecan (SN-38), an inhibitor of topoisomerase 1, is inactive after being subjected to hepatic glucuronidation, which results in SN-38G, which is subsequently eliminated by bile. However once in the gut lumen, SN-38G is reactivated to SN-38 by the bacterial β-glucuronidases. According to recent research, β-glucuronidase-producing bacteria including *Faecalibacterium prausnitzii* and *Clostridium* spp. contribute to the gut’s buildup of the active irinotecan metabolite (SN-38) [163].

#### Reduced gut community

Intestinal health and symbiosis between the host and microbiota are both maintained in large part by the composition of the bacterial community [25]. It has been reported that the microbial community is reciprocally modified by anticancer drugs. *Anaerobes* and *streptococci* were both absolute and relative microbial abundance decreasers, whereas *Bacteroides* were relative microbial abundance increasers, according to a research done on rats given methotrexate. Reduced villous length and diarrhea were linked to this alteration in microbial composition [164]. Additionally, a clinical study on 28 non-Hodgkin lymphoma patients who received a 5-day myeloablative chemotherapy regimen revealed profound changes in the gut microbial community’s structure and reduction of its diversity, as well as an increase in the abundance of Proteobacteria and a decrease in Firmicutes and Actinobacteria [165]. After chemotherapy, there was a decrease in taxa that have been demonstrated to suppress inflammation by altering the NF-κB pathway and by producing SCFAs. Further studies revealed a negative correlation between the quantity of Firmicutes and several metabolic pathways linked to intestinal inflammation, including cell motility, glycan metabolism, and xenobiotic degradation [165].

### Role of gut microbiota in immunotherapy response

Cancer cells exert a variety of immunosuppression mechanisms that prevent antitumor immune responses and enhance tumor escape [166]. Among these mechanisms, cancer immuno-editing is a process tightly orchestrated by immune receptors controlling either the activation or the inhibition of immune responses, called immune checkpoints [167]. Thus, the last decade was marked by a bloom of clinical trials of immunotherapy for cancer treatment [168]. Cancer immunotherapy, based on the sensitization of the patient’s immune system, aims to boost the immune system and regulate the TME for an eventual antitumoral effect, unlike using cytotoxic treatment with chemotherapeutic agents to directly kill the tumor cells [169]. Immunotherapy has attracted attention in recent years as it is significantly advantageous to provide long-lasting anticancer effects to patients who were unresponsive to conventional therapy. In particular, the discovery and application of monoclonal antibodies against immune checkpoints have significantly advanced cancer treatment [170]. Immunological checkpoint blockade is most commonly used to inhibit the immune checkpoint regulators CTLA-4 and PD-1 or its ligand PD-L1 [171]. To date, several ICIs have received Food and Drug Administration (FDA) approval and are now widely indicated in numerous cancer types [168, 172]. In particular, metastatic melanoma and non-small-cell lung cancer are being treated with the use of PD-1 (nivolumab, pembrolizumab, and cemiplimab), PD-L1 (atezolizumab, avelumab, and durvalumab), and CTLA-4 (ipilimumab) blockers [173].

However, the patients’ responses to immunotherapy are often limited by considerable heterogeneity with resistance and immune-related adverse events [174]. Moreover, the efficacy of immune checkpoint inhibitor therapy has been shown only in 10–30% of treated patients [175]. The therapeutic outcomes depend on host and environmental factors, which have been recently connected with gut microbiota. Mounting evidence from preclinical and clinical studies highlighted the important role of gut microbiota in modulating the immune system [176], both local and systemic, the immunological response in anti-tumor activity, and immunotherapy efficacy [177].

#### Effects of the gut microbiota on CTLA-4 therapy

Ipilimumab is a monoclonal antibody that works by blocking CTLA-4, mainly used for the treatment of metastatic melanoma [178]. Emergent data from preclinical studies showed that the efficacy of ipilimumab treatment is affected by gut microbiota modulation and depends particularly on distinct *Bacteroides* species [179]. In fact, in germ-free and antibiotics-treated mice, the response to anti-CTLA-4 treatment was compromised [180]. Besides, the efficacy of CTLA-4 blockade was restored in these *in vivo* models after gavage with *B. fragilis*, where animals responded the best to ipilimumab treatment, and the size of the tumors was negatively correlated with the outgrowth of *B. fragilis* [181]. It has been hypothesized that *B. fragilis* boosts Th1 immune response in lymph nodes that is dependent on interleukin-12 (IL-12) and encourages intratumorous DC maturation, restoring their responsiveness to CTLA-4 inhibition [182]. In other key clinical studies that strongly support the inclusion of microbial targeting in anti-tumor immunotherapy efficacy and complementing the animal models, similar observation has been reported in a good responder group of patients treated with ipilimumab [183]. The analysis of stool samples among this group showed a selective enrichment of *B. fragilis* [183]. In the same context, other species, like *Bifidobacterium breve* and *Bifidobacterium longum* have been correlated to the activation of immune cells in the TME during ipilimumab therapy [184].

#### Effects of the gut microbiota on PD-1/PD-L1 therapy

Similarly, the clinical response to the antitumor monoclonal antibodies against-PD-1 or its ligand PD-L1 was shown to have a direct link with microbiome signature [185]. A recent animal study showed improved efficacy of the anti-PD-1/PD-L1 treatment in germ-free mice transplanted with fecal microbiota from good or poor responders and supplemented with Clostridiales and *Faecalibacterium* species [186]. Another report has demonstrated that the addition of *Akkermansia muciniphila*, *Alistipes indistinctus*, or *E. hirae* could invigorate the immunotherapy treatment in germ-free mice [187, 188]. Accordingly, a metagenomic analysis showed that the enrichment of *Akkermansia muciniphila* in faecal samples from non-small cell lung cancer (NSCLC) and renal cell carcinoma (RCC) patients were related to a promising clinical outcome during anti-PD-1 and anti-PD-L1 therapies [189]. Further, a high level of commensal bacteria in patients with melanoma was correlated with enhanced antitumor immune response. One suggested mechanism to explain the improvement of immunotherapy treatment is an increase in intratumoral infiltration of CD8^+^ T cells with enhanced tumor-specific T cell and DC-mediated immune responses [190]. On the other hand, the reduced response or the resistance to PD-1/PD-L1 immunotherapy has been associated with a high abundance of Bacteroidales [191]. Nevertheless, numerous studies pointed out the impact of antibiotics to engender dysbiosis that subsequently compromise the anti-PD-1 efficacy in cancer patients [192]. For instance, across the different cohort studies related to the anti-PD-1 or PD-L1 therapies, an overlap in the involved microbiota species has been raised. The heterogeneity of cancer types, the tumor’s microenvironment, the host genetic background and immune system, or even the sequencing approaches, may lead to biases [192]. It seems likely that a microbial responder profile reflects a combination of key species instead of a separate one.

When considered collectively, those results strongly imply that a balance between “useful” and “non-beneficial” bacteria may be able to influence the effectiveness of both immunotherapy and chemotherapeutic treatments. An anticancer medication has the potential to change the gut microbiota’s composition and the supplementation of beneficial bacterial strains may lead to better efficacy and response of the anticancer therapy.

## Targeting microbiota as a promising tool to optimise anti-cancer therapy response

It is conceivable to target the gut microbiota as a therapeutic strategy given its crucial involvement in cancer therapy. Recently, few studies explored microbiota modulation to improve cancer therapies response, however, the mechanisms remain less understood [193]. In the current section of the review, we concentrated on prospective methods for modifying the microbiota to increase therapeutic effectiveness.

### Probiotics in oncology

The cornerstones of modern anti-cancer therapies, including chemotherapy, targeted therapy, immunotherapy, and radiation, can have a variety of, sometimes severe, adverse effects on patients [194–196]. In order to assess the probiotics’ overall effectiveness in reducing the risk and severity of such anti-cancer treatment-related toxicity, a number of preclinical and clinical investigations have been conducted [4]. In fact, the administration of probiotics to cancer patients, mainly *Lactobacilli*, is to re-populate the altered patients’ gut microbiota, thus re-establishing the composition and functionality of the commensal bacteria [197]. Probiotics are typically viewed as harmless, however, giving them to immunocompromised cancer patients runs the danger of passing on antibiotic resistance and opportunistic infections [198]. Despite this, probiotics have been proven to be effective in treating diarrhea and other gut-related side effects after anti-cancer treatment, leading to the restoration of a balanced intestinal microbiota [199]. The combination of the two probiotic species *Lactobacillus johnsonii* and *Bifidobacterium longum* was given to cancer patients for the first time in 2010. Researchers discovered that *Lactobacillus johnsonii* was able to adhere to the colonic mucosa, lowering the concentration of gut pathogens and modifying the local immune system [200]. In a clinical randomized control study, patients treated for a nasopharyngeal carcinoma received a combination of probiotics which resulted in an enhanced immune response and a decreased rate of oral mucositis after chemoradiotherapy. Indeed, the probiotic combination has been shown to increase the number of CD4^+^ T cells, CD8^+^ T cells, and CD3^+^ T cells [201].

Viaud et al. [155] found that tumor-bearing mice treated with CTX had dysbiosis, which was a breakdown of gut mucosal integrity related to chemotherapy. In mice given antibiotic treatment, they observed a decreased CTX-induced Th17 cell conversion and poorer tumor remission. But oral administration of *E. hirae* and *Lactobacillus johnsonii* to these mice restored the anti-tumor effectiveness of CTX by enhancing the T cell immune response [155]. Altogether, this study highlights that *E. hirae* and *Lactobacillus johnsonii* could be used as probiotics in combination with CTX therapy to improve treatment efficacy in cancer patients.

### Use of prebiotics and synbiotics in oncology

Good evidence in both animal and human studies supports that dietary fibers known as prebiotics could be metabolized by gut bacteria leading to an increase in the colonization and relative growth of particular bacteria and their metabolites, which may improve the anti-tumor treatment effect. The presence of certain bacteria in the human gut has been proven to be necessary for prebiotics to have any possible impact. In order to cure cancer, synbiotics, a mix of probiotics and prebiotics, appear to be helpful. According to recent research, decreased fiber intake lowered the amount of SCFA that the microbiota generated and increased the use of less-preferred substrates including amino acids and host mucins [202]. PD-1 blockade medication is currently being used to treat melanoma patients, and clinical investigations have shown that increases in bacterial diversity brought about by high-fiber diets significantly enhanced progression-free survival [203]. To firmly support the efficacy and safety of giving probiotics, prebiotics, and synbiotics during or after anti-cancer therapy, more preclinical and clinical studies may be required.

### FMT

Due to its potential therapeutic value, the 4th-century Chinese idea of FMT has lately attracted attention from researchers in biology and clinical medicine [204]. FMT involves the transplantation of stool collected from a healthy donor into the intestinal tract of a patient with an altered intestinal microbiome. FMT was originally utilized to control host metabolism in obese people, outside of the setting of oncologic illnesses [205]. Indeed, the transplant of the FMT from lean donors to obese recipients has resulted in variable improvements in the insulin-sensitivity which was associated with an increased abundance of butyrate-producing intestinal microbes [206]. Interestingly, the transfer of FMT from good responders to anticancer treatment to mouse models has been reported to enhance treatment response in these animals. In fact, the study found that mice getting FMT from treatment strong responders had a larger proportion of CD8^+^ T cells and cells expressing CD45^+^, CD11b^+^, and lymphocyte antigen 6 complex, locus G (Ly6G^+^) and a lower level of myeloid cells showing CD11b^+^ and CD11c^+^ [207], suggesting a significant enrichment of adaptative and innate effector cells and a decrease of cells with suppressive activity. Further, anticancer immunity was suppressed in receivers of fecal material from poor responders because they had larger amounts of CD4^+^, IL-17^+^, Th17 cells and CD4^+^, forkhead box P3 (FoxP3^+^) Tregs in the spleen [207]. Decrease in the number of CD4^+^ FoxP3^+^ Treg cells with suppressive activity and an increase in the activity of CD8^+^ cytotoxic T cells. In line with this context, it has been proposed that the primary role of neutrophils in promoting antitumor immunity is to control IL-17 secretion and thus indirectly suppress tumor growth by promoting CD8^+^ T cell function [207].

Additionally, recipient germ free (GF) mice were given feces from three responders and three non-responders, followed by B16 implantation. Receivers of fecal material from responding patients showed increased tumor control, boosted T cell responses, and improved anti-PD-L1 treatment effectiveness in SIY melanoma cells. Two of three mouse cohorts implanted with responder feces showed slower tumor growth, whereas two mice transplanted with non-responder feces showed quicker baseline tumor growth [208]. Collectively, these results revealed that a “favorable” gut microbiome may enhance systemic and anti-tumor immune responses.

According to several clinical investigations, fecal treatment may be a potential approach in treating a variety of human ailments as FMT is used more frequently.

### Fecal virome transplants

A novel therapeutic intervention has been recently developed known as fecal virome transplantation (FVT), where only the viral component from feces is transplanted [209, 210]. A study conducted by Draper et al. [211] showed that FVT mainly consisted of phages, and improved antibiotic-induced bacterial dysbiosis. The transplanted phages have shown to be able to colonize the gut and reshape the bacteriome similarly to a pre-antibiotic state. In FVT studies conducted in murine models [210], the gut viral composition was shown to be dominated by the order Caudovirales and the family Microviridae viruses. In another study, treatment with lytic and temperate gut phages modulated gut microbial composition. In fact, they demonstrated that lytic phages enhanced the beneficial species of gut microbiota, and temperate phages stimulated the growth of commensal in the gut [209]. More profoundly, phages are not only able to modulate the microbiome but also its associated metabolome [212]. Indeed, Hsu et al. [212] revealed that gnotobiotic mice were subjected to lytic phages after they were colonized with commensals. These findings suggested that phage-led microbiome modulation was indeed due to intense microbe–microbe (intra- and inter-microbial) interactions which resulted in remarkable changes in the metabolome.

## Conclusions

Huge efforts are being made today to better understand the gut microbiota’s involvement in influencing how the body reacts to cancer treatments as well as the dynamic interplay between the host and gut microbiota. In the current review, we highlight the critical role of gut microbiota to modulate the course of carcinogenes and the immune response, impacting therefore the efficacy of cancer chemotherapy and immunotherapy. Dysbiosis may affect the path of carcinogenesis because microbial activities seem to have an impact on epithelial DNA damage and epigenetic modifications that result in dysplasia, clonal expansion, and malignant transformation. The dysbiotic microbiota promotes tumorigenesis in a number of ways, such as by directly activating bacterial toxins like ROS which is involved in DNA damage, *CagA*, and *VacA* that interfere with the proliferation and apoptosis pathways or by indirectly activating inflammatory pathways induced by microbe-associated molecular pattern (MAMP) activating TLR and other PPR. Interestingly, distinct beneficial bacterial species were suggested to improve the antitumor effect and to characterize good responders to cancer therapy. It has been suggested that the microbiota may exert its preventative effects by rendering carcinogens inactive and producing SCFAs, such as butyrate, and propionate. However, the exact mechanisms underlying this crosstalk remain elusive and merit a more comprehensive understanding. Besides, valuable works on the modulation of gut microbiota, based on prebiotics/probiotics/FMT, have shown promising improvement in cancer therapy response. These strategies have demonstrated promising results through a variety of mechanisms, including altering the microbiota composition, modulating the innate and adaptative immune responses, improving gut barrier function, preventing pathogen colonization, and exerting selective cytotoxicity against tumor cells. However, it should be noted that they come with drawbacks and controversies that may result in clinical problems. Although this innovative field is still limited, targeting microbiota might provide novel therapeutic strategies to treat cancers.

## Abbreviations

*B. fragilis*: *Bacteroides fragilis*
BCs: breast cancers
*C. elegans*: *Caenorhabditis elegans*
*CagA*: cytotoxin-associated gene A
CRC: colorectal cancer
CTLA-4: cytotoxic T lymphocyte-associated antigen-4
CTX: cyclophosphamide
*E. coli*: *Escherichia coli*
*E. hirae*: *Enterococcus hirae*
EBV: Epstein-Barr virus
*F. nucleatum*: *Fusobacterium nucleatum*
FMT: fecal microbiota transplantation
FVT: fecal virome transplantation
GI: gastrointestinal
*H. pylori*: *Helicobacter pylori*
HPV: human papillomaviruses
HSV1/2: herpes simplex virus 1/2
LMP1: latent membrane protein 1
MMR: measles, mumps, and rubella
PD-1: programmed cell death protein-1
PD-L1: programmed cell death protein-1-ligand 1
ROS: reactive oxygen species
SCFA: short-chain fatty acids
TME: tumor microenvironment
Treg: regulatory T cell
*VacA*: vacuolating cytotoxin gene A

## Declarations

### Copyright

© The Author(s) 2023.

## References

[1] Vasan N Baselga J Hyman DM. A view on drug resistance in cancer. Nature. 2019;575:299–309. 10.1038/s41586-019-1730-1 31723286 PMC8008476

[2] Ellis LM Hicklin DJ. Resistance to targeted therapies: refining anticancer therapy in the era of molecular oncology. Clin Cancer Res. 2009;15:7471–8. 10.1158/1078-0432.CCR-09-1070 20008847

[3] Foo J Michor F. Evolution of acquired resistance to anti-cancer therapy. J Theor Biol. 2014;355:10–20. 10.1016/j.jtbi.2014.02.025 24681298 PMC4058397

[4] Alexander JL Wilson ID Teare J Marchesi JR Nicholson JK Kinross JM. Gut microbiota modulation of chemotherapy efficacy and toxicity. Nat Rev Gastroenterol Hepatol. 2017;14:356–65. 10.1038/nrgastro.2017.20 28270698

[5] Huang J Jiang Z Wang Y Fan X Cai J Yao X Modulation of gut microbiota to overcome resistance to immune checkpoint blockade in cancer immunotherapy. Curr Opin Pharmacol. 2020;54:1–10. 10.1016/j.coph.2020.06.004 32619934

[6] Garajová I Balsano R Wang H Leonardi F Giovannetti E Deng D The role of the microbiome in drug resistance in gastrointestinal cancers. Expert Rev Anticancer Ther. 2021;21:165–76. 10.1080/14737140.2021.1844007 33115280

[7] Shui L Yang X Li J Yi C Sun Q Zhu H. Gut microbiome as a potential factor for modulating resistance to cancer immunotherapy. Front Immunol. 2020;10:2989. 10.3389/fimmu.2019.02989 32010123 PMC6978681

[8] Weersma RK Zhernakova A Fu J. Interaction between drugs and the gut microbiome. Gut. 2020;69:1510–9. 10.1136/gutjnl-2019-320204 32409589 PMC7398478

[9] Villéger R Lopès A Carrier G Veziant J Billard E Barnich N Intestinal microbiota: a novel target to improve anti-tumor treatment? Int J Mol Sci. 2019;20:4584. 10.3390/ijms20184584 31533218 PMC6770123

[10] Gori S Inno A Belluomini L Bocus P Bisoffi Z Russo A Gut microbiota and cancer: how gut microbiota modulates activity, efficacy and toxicity of antitumoral therapy. Crit Rev Oncol Hematol. 2019;143:139–47. 10.1016/j.critrevonc.2019.09.003 31634731

[11] Campbell SC Wisniewski PJ 2nd. Exercise is a novel promoter of intestinal health and microbial diversity. Exerc Sport Sci Rev. 2017;45:41–7. 10.1249/JES.0000000000000096 27782912

[12] Pushalkar S Hundeyin M Daley D Zambirinis CP Kurz E Mishra A The pancreatic cancer microbiome promotes oncogenesis by induction of innate and adaptive immune suppression. Cancer Discov. 2018;8:403–16. Erratum in: Cancer Discov. 2020;10:1988. 10.1158/2159-8290.CD-17-1134 29567829 PMC6225783

[13] Riquelme E Zhang Y Zhang L Montiel M Zoltan M Dong W Tumor microbiome diversity and composition influence pancreatic cancer outcomes. Cell. 2019;178:795–806.E12. 10.1016/j.cell.2019.07.008 31398337 PMC7288240

[14] Yu J Feng Q Wong SH Zhang D Liang QY Qin Y Metagenomic analysis of faecal microbiome as a tool towards targeted non-invasive biomarkers for colorectal cancer. Gut. 2017;66:70–8. 10.1136/gutjnl-2015-309800 26408641

[15] Moore WEC Holdeman LV. Human fecal flora: the normal flora of 20 Japanese-Hawaiians. Appl Microbiol. 1974;27:961–79. 10.1128/am.27.5.961-979.1974 4598229 PMC380185

[16] Marchesi JR Adams DH Fava F Hermes GD Hirschfield GM Hold G The gut microbiota and host health: a new clinical frontier. Gut. 2016;65:330–9. 10.1136/gutjnl-2015-309990 26338727 PMC4752653

[17] Chen Z Zhu S Xu G. Targeting gut microbiota: a potential promising therapy for diabetic kidney disease. Am J Transl Res. 2016;8:4009–16. Erratum in: Am J Transl Res. 2018;10:333. 27829988 PMC5095297

[18] Yamashiro Y. Gut microbiota in health and disease. Ann Nutr Metab. 2017;71:242–6. 10.1159/000481627 29136611

[19] Ogilvie LA Jones BV. The human gut virome: a multifaceted majority. Front Microbiol. 2015;6:918. 10.3389/fmicb.2015.00918 26441861 PMC4566309

[20] Shkoporov AN Hill C. Bacteriophages of the human gut: the “Known Unknown” of the microbiome. Cell Host Microbe. 2019;25:195–209. 10.1016/j.chom.2019.01.017 30763534

[21] Dethlefsen L Eckburg PB Bik EM Relman DA. Assembly of the human intestinal microbiota. Trends Ecol Evol. 2006;21:517–23. 10.1016/j.tree.2006.06.013 16820245

[22] Human Microbiome Project Consortium. Structure, function and diversity of the healthy human microbiome. Nature. 2012;486:207–14. 10.1038/nature11234 22699609 PMC3564958

[23] O’Hara AM Shanahan F. The gut flora as a forgotten organ. EMBO Rep. 2006;7:688–93. 10.1038/sj.embor.7400731 16819463 PMC1500832

[24] Natividad JM Verdu EF. Modulation of intestinal barrier by intestinal microbiota: pathological and therapeutic implications. Pharmacol Res. 2013;69:42–51. 10.1016/j.phrs.2012.10.007 23089410

[25] Upadhyaya S Banerjee G. Type 2 diabetes and gut microbiome: at the intersection of known and unknown. Gut Microbes. 2015;6:85–92. 10.1080/19490976.2015.1024918 25901889 PMC4615359

[26] LeBlanc JG Milani C de Giori GS Sesma F van Sinderen D Ventura M. Bacteria as vitamin suppliers to their host: a gut microbiota perspective. Curr Opin Biotechnol. 2013;24:160–8. 10.1016/j.copbio.2012.08.005 22940212

[27] Chow J Lee SM Shen Y Khosravi A Mazmanian SK. Chapter 8 - host–bacterial symbiosis in health and disease. In: Fagarasan S Cerutti A, editors. Advances in immunology. Academic Press; 2010. pp. 243–74. 10.1016/B978-0-12-381300-8.00008-3 PMC3152488 21034976

[28] Carding S Verbeke K Vipond DT Corfe BM Owen LJ. Dysbiosis of the gut microbiota in disease. Microb Ecol Health Dis. 2015;26:26191. 10.3402/mehd.v26.26191 25651997 PMC4315779

[29] Federico A Dallio M DI Sarno R Giorgio V Miele L. Gut microbiota, obesity and metabolic disorders. Minerva Gastroenterol Dietol. 2017;63:337–44. 10.23736/S1121-421X.17.02376-5 28927249

[30] Gurung M Li Z You H Rodrigues R Jump DB Morgun A Role of gut microbiota in type 2 diabetes pathophysiology. EBioMedicine. 2020;51:102590. 10.1016/j.ebiom.2019.11.051 31901868 PMC6948163

[31] Khan I Ullah N Zha L Bai Y Khan A Zhao T Alteration of gut microbiota in inflammatory bowel disease (IBD): cause or consequence? IBD treatment targeting the gut microbiome. Pathogens. 2019;8:126. 10.3390/pathogens8030126 31412603 PMC6789542

[32] Vivarelli S Salemi R Candido S Falzone L Santagati M Stefani S Gut microbiota and cancer: from pathogenesis to therapy. Cancers (Basel). 2019;11:38. 10.3390/cancers11010038 30609850 PMC6356461

[33] Zitvogel L Daillère R Roberti MP Routy B Kroemer G. Anticancer effects of the microbiome and its products. Nat Rev Microbiol. 2017;15:465–78. 10.1038/nrmicro.2017.44 28529325

[34] Takeshima H Ushijima T. Accumulation of genetic and epigenetic alterations in normal cells and cancer risk. NPJ Precis Oncol. 2019;3:7. 10.1038/s41698-019-0079-0 30854468 PMC6403339

[35] Weir B Zhao X Meyerson M. Somatic alterations in the human cancer genome. Cancer Cell. 2004;6:433–8. 10.1016/j.ccr.2004.11.004 15542426

[36] Barnes JL Zubair M John K Poirier MC Martin FL. Carcinogens and DNA damage. Biochem Soc Trans. 2018;46:1213–24. 10.1042/BST20180519 30287511 PMC6195640

[37] Irigaray P Newby JA Clapp R Hardell L Howard V Montagnier L Lifestyle-related factors and environmental agents causing cancer: an overview. Biomed Pharmacother. 2007;61:640–58. 10.1016/j.biopha.2007.10.006 18055160

[38] Cohen SM Ellwein LB. Cell proliferation in carcinogenesis. Science. 1990;249:1007–11. 10.1126/science.2204108 2204108

[39] Comings DE. A general theory of carcinogenesis. Proc Natl Acad Sci U S A. 1973;70:3324–8. 10.1073/pnas.70.12.3324 4202843 PMC427229

[40] Devi PU. Basics of carcinogenesis. Health Adm. 2004;17:16–24.

[41] Chakravarthi BV Nepal S Varambally S. Genomic and epigenomic alterations in cancer. Am J Pathol. 2016;186:1724–35. 10.1016/j.ajpath.2016.02.023 27338107 PMC4929396

[42] Di Nicolantonio F Arena S Tabernero J Grosso S Molinari F Macarulla T Deregulation of the PI3K and KRAS signaling pathways in human cancer cells determines their response to everolimus. J Clin Invest. 2010;120:2858–66. 10.1172/JCI37539 20664172 PMC2912177

[43] Chen B Li H Zeng X Yang P Liu X Zhao X Roles of microRNA on cancer cell metabolism. J Transl Med. 2012;10:228. 10.1186/1479-5876-10-228 23164426 PMC3563491

[44] Khan Z Bisen PS. Oncoapoptotic signaling and deregulated target genes in cancers: special reference to oral cancer. Biochim Biophys Acta Rev Cancer. 2013;1836:123–45. 10.1016/j.bbcan.2013.04.002 23602834

[45] Vaupel P Mayer A. Hypoxia in cancer: significance and impact on clinical outcome. Cancer Metastasis Rev. 2007;26:225–39. 10.1007/s10555-007-9055-1 17440684

[46] Jun JC Rathore A Younas H Gilkes D Polotsky VY. Hypoxia-inducible factors and cancer. Curr Sleep Med Rep. 2017;3:1–10. 10.1007/s40675-017-0062-7 28944164 PMC5607450

[47] Dongre A Weinberg RA. New insights into the mechanisms of epithelial-mesenchymal transition and implications for cancer. Nat Rev Mol Cell Biol. 2019;20:69–84. 10.1038/s41580-018-0080-4 30459476

[48] Ribatti D Tamma R Annese T. Epithelial-mesenchymal transition in cancer: a historical overview. Transl Oncol. 2020;13:100773. 10.1016/j.tranon.2020.100773 32334405 PMC7182759

[49] Hanahan D Weinberg RA. Hallmarks of cancer: the next generation. Cell. 2011;144:646–74. 10.1016/j.cell.2011.02.013 21376230

[50] Weisburger JH Reddy BS Narisawa T Wynder EL. Germ-free status and colon tumor induction by N-Methyl-N’-Nitro-N-Nitrosoguanidine. Proc Soc Exp Biol Med. 1975;148:1119–21. 10.3181/00379727-148-38700 1129327

[51] Li Y Kundu P Seow SW de Matos CT Aronsson L Chin KC Gut microbiota accelerate tumor growth via c-jun and STAT3 phosphorylation in APC ^Min/+^ mice. Carcinogenesis. 2012;33:1231–8. 10.1093/carcin/bgs137 22461519

[52] Gupta A Madani R Mukhtar H. Streptococcus bovis endocarditis, a silent sign for colonic tumour. Colorectal Dis. 2010;12:164–71. 10.1111/j.1463-1318.2009.01814.x 19226366

[53] Kostic AD Gevers D Pedamallu CS Michaud M Duke F Earl AM Genomic analysis identifies association of *Fusobacterium* with colorectal carcinoma. Genome Res. 2012;22:292–8. 10.1101/gr.126573.111 22009990 PMC3266036

[54] Viljoen KS Dakshinamurthy A Goldberg P Blackburn JM. Quantitative profiling of colorectal cancer-associated bacteria reveals associations between *Fusobacterium* spp., enterotoxigenic *bacteroides fragilis* (ETBF) and clinicopathological features of colorectal cancer. PLoS One. 2015;10:e0119462. 10.1371/journal.pone.0119462 25751261 PMC4353626

[55] Gao Z Guo B Gao R Zhu Q Qin H. Microbiota disbiosis is associated with colorectal cancer. Front Microbiol. 2015;6:20. 10.3389/fmicb.2015.00020 25699023 PMC4313696

[56] Buc E Dubois D Sauvanet P Raisch J Delmas J Darfeuille-Michaud A High prevalence of mucosa-associated *E. coli* producing cyclomodulin and genotoxin in colon cancer. PLoS One. 2013;8:e56964. 10.1371/journal.pone.0056964 23457644 PMC3572998

[57] Castellarin M Warren RL Freeman JD Dreolini L Krzywinski M Strauss J *Fusobacterium nucleatum* infection is prevalent in human colorectal carcinoma. Genome Res. 2012;22:299–306. 10.1101/gr.126516.111 22009989 PMC3266037

[58] Wang T Cai G Qiu Y Fei N Zhang M Pang X Structural segregation of gut microbiota between colorectal cancer patients and healthy volunteers. ISME J. 2012;6:320–9. 10.1038/ismej.2011.109 21850056 PMC3260502

[59] Ahn J Sinha R Pei Z Dominianni C Wu J Shi J Human gut microbiome and risk for colorectal cancer. J Natl Cancer Inst. 2013;105:1907–11. 10.1093/jnci/djt300 24316595 PMC3866154

[60] Chen W Liu F Ling Z Tong X Xiang C. Human intestinal lumen and mucosa-associated microbiota in patients with colorectal cancer. PLoS One. 2012;7:e39743. 10.1371/journal.pone.0039743 22761885 PMC3386193

[61] Magat EM Balanag GA CariÑo AM Fellizar A Ortin TS Guevarra L Jr *Clostridioides difficile* antibody response of colorectal cancer patients versus clinically healthy individuals. Biosci Microbiota Food Health. 2020;39:123–7. 10.12938/bmfh.2020-010 32775130 PMC7392905

[62] Flemer B Lynch DB Brown JM Jeffery IB Ryan FJ Claesson MJ Tumour-associated and non-tumour-associated microbiota in colorectal cancer. Gut. 2017;66:633–43. 10.1136/gutjnl-2015-309595 26992426 PMC5529966

[63] Handa O Naito Y Yoshikawa T. *Helicobacter pylori*: a ROS-inducing bacterial species in the stomach. Inflamm Res. 2010;59:997–1003. 10.1007/s00011-010-0245-x 20820854

[64] Gagnière J Raisch J Veziant J Barnich N Bonnet R Buc E Gut microbiota imbalance and colorectal cancer. World J Gastroenterol. 2016;22:501–18. 10.3748/wjg.v22.i2.501 26811603 PMC4716055

[65] Nakatsu G Li X Zhou H Sheng J Wong SH Wu WK Gut mucosal microbiome across stages of colorectal carcinogenesis. Nat Commun. 2015;6:8727. 10.1038/ncomms9727 26515465 PMC4640069

[66] Bonnet M Buc E Sauvanet P Darcha C Dubois D Pereira B Colonization of the human gut by *E. coli* and colorectal cancer risk. Clin Cancer Res. 2014;20:859–67. 10.1158/1078-0432.CCR-13-1343 24334760

[67] Kohoutova D Smajs D Moravkova P Cyrany J Moravkova M Forstlova M *Escherichia coli* strains of phylogenetic group B2 and D and bacteriocin production are associated with advanced colorectal neoplasia. BMC Infect Dis. 2014;14:733. 10.1186/s12879-014-0733-7 25540872 PMC4300055

[68] Flanagan L Schmid J Ebert M Soucek P Kunicka T Liska V *Fusobacterium nucleatum* associates with stages of colorectal neoplasia development, colorectal cancer and disease outcome. Eur J Clin Microbiol Infect Dis. 2014;33:1381–90. 10.1007/s10096-014-2081-3 24599709

[69] Ito M Kanno S Nosho K Sukawa Y Mitsuhashi K Kurihara H Association of *Fusobacterium nucleatum* with clinical and molecular features in colorectal serrated pathway. Int J Cancer. 2015;137:1258–68. 10.1002/ijc.29488 25703934

[70] Tahara T Yamamoto E Suzuki H Maruyama R Chung W Garriga J *Fusobacterium* in colonic flora and molecular features of colorectal carcinoma. Cancer Res. 2014;74:1311–8. 10.1158/0008-5472.CAN-13-1865 24385213 PMC4396185

[71] Teng J Zhao Y Jiang Y Wang Q Zhang Y. Correlation between gut microbiota and lung cancer. Zhongguo Fei Ai Za Zhi. 2020;23:909–15. Chinese. 10.3779/j.issn.1009-3419.2020.101.39 32798442 PMC7583874

[72] Zhao Y Liu Y Li S Peng Z Liu X Chen J Role of lung and gut microbiota on lung cancer pathogenesis. J Cancer Res Clin Oncol. 2021;147:2177–86. 10.1007/s00432-021-03644-0 34018055 PMC8236441

[73] Fernández MF Reina-Pérez I Astorga JM Rodríguez-Carrillo A Plaza-Díaz J Fontana L. Breast cancer and its relationship with the microbiota. Int J Environ Res Public Health. 2018;15:1747. 10.3390/ijerph15081747 30110974 PMC6121903

[74] Chen J Douglass J Prasath V Neace M Atrchian S Manjili MH The microbiome and breast cancer: a review. Breast Cancer Res Treat. 2019;178:493–6. 10.1007/s10549-019-05407-5 31456069

[75] Toumazi D El Daccache S Constantinou C. An unexpected link: the role of mammary and gut microbiota on breast cancer development and management (Review). Oncol Rep. 2021;45:80. 10.3892/or.2021.8031 33786630

[76] Samkari AA Alsulami M Bataweel L Altaifi R Altaifi A Saleem AM Body microbiota and its relationship with benign and malignant breast tumors: a systematic review. Cureus. 2022;14:e25473. 10.7759/cureus.25473 35783895 PMC9240997

[77] Tjalsma H Boleij A Marchesi JR Dutilh BE. A bacterial driver-passenger model for colorectal cancer: beyond the usual suspects. Nat Rev Microbiol. 2012;10:575–82. 10.1038/nrmicro2819 22728587

[78] Cuevas-Ramos G Petit CR Marcq I Boury M Oswald E Nougayrède JP. *Escherichia coli* induces DNA damage *in vivo* and triggers genomic instability in mammalian cells. Proc Natl Acad Sci U S A. 2010;107:11537–42. 10.1073/pnas.1001261107 20534522 PMC2895108

[79] Nešić D Hsu Y Stebbins CE. Assembly and function of a bacterial genotoxin. Nature. 2004;429:429–33. 10.1038/nature02532 15164065

[80] Huycke MM Moore DR. *In vivo* production of hydroxyl radical by *Enterococcus faecalis* colonizing the intestinal tract using aromatic hydroxylation. Free Radic Biol Med. 2002;33:818–26. 10.1016/S0891-5849(02)00977-2 12208369

[81] Arthur JC Perez-Chanona E Mühlbauer M Tomkovich S Uronis JM Fan TJ Intestinal inflammation targets cancer-inducing activity of the microbiota. Science. 2012;338:120–3. 10.1126/science.1224820 22903521 PMC3645302

[82] Cooke MS Evans MD Dizdaroglu M Lunec J. Oxidative DNA damage: mechanisms, mutation, and disease. FASEB J. 2003;17:1195–214. 10.1096/fj.02-0752rev 12832285

[83] Lundberg JO Weitzberg E Cole JA Benjamin N. Nitrate, bacteria and human health. Nat Rev Microbiol. 2004;2:593–602. Erratum in: Nat Rev Microbiol. 2004;2:681. 10.1038/nrmicro929 15197394

[84] Maddocks OD Scanlon KM Donnenberg MS. An *Escherichia coli* effector protein promotes host mutation via depletion of DNA mismatch repair proteins. mBio. 2013;4:e00152–13. 10.1128/mBio.00152-13 23781066 PMC3684829

[85] Maddocks ODK Short AJ Donnenberg MS Bader S Harrison DJ. Attaching and effacing *Escherichia coli* downregulate DNA mismatch repair protein *in vitro* and are associated with colorectal adenocarcinomas in humans. PLoS One. 2009;4:e5517. 10.1371/journal.pone.0005517 19436735 PMC2677459

[86] Nejati S Karkhah A Darvish H Validi M Ebrahimpour S Nouri HR. Influence of *Helicobacter pylori* virulence factors CagA and VacA on pathogenesis of gastrointestinal disorders. Microb Pathog. 2018;117:43–8. 10.1016/j.micpath.2018.02.016 29432909

[87] Ohnishi N Yuasa H Tanaka S Sawa H Miura M Matsui A Transgenic expression of *Helicobacter pylori* CagA induces gastrointestinal and hematopoietic neoplasms in mouse. Proc Natl Acad Sci U S A. 2008;105:1003–8. 10.1073/pnas.0711183105 18192401 PMC2242726

[88] Yong X Tang B Li BS Xie R Hu CJ Luo G *Helicobacter pylori* virulence factor CagA promotes tumorigenesis of gastric cancer *via* multiple signaling pathways. Cell Commun Signal. 2015;13:30. 10.1186/s12964-015-0111-0 26160167 PMC4702319

[89] Foegeding NJ Caston RR McClain MS Ohi MD Cover TL. An overview of *Helicobacter pylori* VacA toxin biology. Toxins (Basel). 2016;8:173. 10.3390/toxins8060173 27271669 PMC4926140

[90] Sears CL. Enterotoxigenic *Bacteroides fragilis*: a rogue among symbiotes. Clin Microbiol Rev. 2009;22:349–69. 10.1128/CMR.00053-08 19366918 PMC2668231

[91] Wu S Shin J Zhang G Cohen M Franco A Sears CL. The *Bacteroides fragilis* toxin binds to a specific intestinal epithelial cell receptor. Infect Immun. 2006;74:5382–90. 10.1128/IAI.00060-06 16926433 PMC1594844

[92] Manichanh C Borruel N Casellas F Guarner F. The gut microbiota in IBD. Nat Rev Gastroenterol Hepatol. 2012;9:599–608. 10.1038/nrgastro.2012.152 22907164

[93] Chassaing B Darfeuille-Michaud A. The commensal microbiota and enteropathogens in the pathogenesis of inflammatory bowel diseases. Gastroenterology. 2011;140:1720–8. 10.1053/j.gastro.2011.01.054 21530738

[94] Raisch J Rolhion N Dubois A Darfeuille-Michaud A Bringer MA. Intracellular colon cancer-associated *Escherichia coli* promote protumoral activities of human macrophages by inducing sustained COX-2 expression. Lab Invest. 2015;95:296–307. 10.1038/labinvest.2014.161 25545478

[95] Darfeuille-Michaud A Boudeau J Bulois P Neut C Glasser AL Barnich N High prevalence of adherent-invasive *Escherichia coli* associated with ileal mucosa in Crohn’s disease. Gastroenterology. 2004;127:412–21. 10.1053/j.gastro.2004.04.061 15300573

[96] Louis P Hold GL Flint HJ. The gut microbiota, bacterial metabolites and colorectal cancer. Nat Rev Microbiol. 2014;12:661–72. 10.1038/nrmicro3344 25198138

[97] Schwabe RF Jobin C. The microbiome and cancer. Nat Rev Cancer. 2013;13:800–12. 10.1038/nrc3610 24132111 PMC3986062

[98] Boleij A Tjalsma H. Gut bacteria in health and disease: a survey on the interface between intestinal microbiology and colorectal cancer. Biol Rev Camb Philos Soc. 2012;87:701–30. 10.1111/j.1469-185X.2012.00218.x 22296522

[99] Balkwill FR Capasso M Hagemann T. The tumor microenvironment at a glance. J Cell Sci. 2012;125:5591–6. 10.1242/jcs.116392 23420197

[100] Gupta S Roy A Dwarakanath BS. Metabolic cooperation and competition in the tumor microenvironment: implications for therapy. Front Oncol. 2017;7:68. 10.3389/fonc.2017.00068 28447025 PMC5388702

[101] Kovács T Mikó E Ujlaki G Sári Z Bai P. The microbiome as a component of the tumor microenvironment. Adv Exp Med Biol. 2020;1225:137–53. 10.1007/978-3-030-35727-6_10 32030653

[102] Plottel CS Blaser MJ. Microbiome and malignancy. Cell Host Microbe. 2011;10:324–35. 10.1016/j.chom.2011.10.003 22018233 PMC3264051

[103] Wei MQ Ellem KA Dunn P West MJ Bai CX Vogelstein B. Facultative or obligate anaerobic bacteria have the potential for multimodality therapy of solid tumours. Eur J Cancer. 2007;43:490–6. 10.1016/j.ejca.2006.10.005 17113280

[104] Baban CK Cronin M O’Hanlon D O’Sullivan GC Tangney M. Bacteria as vectors for gene therapy of cancer. Bioeng Bugs. 2010;1:385–94. 10.4161/bbug.1.6.13146 21468205 PMC3056088

[105] Syed Khaja AS Toor SM El Salhat H Faour I Ul Haq N Ali BR Preferential accumulation of regulatory T cells with highly immunosuppressive characteristics in breast tumor microenvironment. Oncotarget. 2017;8:33159–71. 10.18632/oncotarget.16565 28388539 PMC5464858

[106] Vojinovic D Radjabzadeh D Kurilshikov A Amin N Wijmenga C Franke L Relationship between gut microbiota and circulating metabolites in population-based cohorts. Nat Commun. 2019;10:5813. 10.1038/s41467-019-13721-1 31862950 PMC6925111

[107] Poore GD Kopylova E Zhu Q Carpenter C Fraraccio S Wandro S Microbiome analyses of blood and tissues suggest cancer diagnostic approach. Nature. 2020;579:567–74. 10.1038/s41586-020-2095-1 32214244 PMC7500457

[108] Hang S Paik D Yao L Kim E Trinath J Lu J Bile acid metabolites control TH17 and Treg cell differentiation. Nature. 2019;576:143–8. Erratum in: Nature. 2020;579:E7. 10.1038/s41586-019-1785-z 31776512 PMC6949019

[109] Fluckiger A Daillère R Sassi M Sixt BS Liu P Loos F Cross-reactivity between tumor MHC class I-restricted antigens and an enterococcal bacteriophage. Science. 2020;369:936–42. 10.1126/science.aax0701 32820119

[110] Mager LF Burkhard R Pett N Cooke NCA Brown K Ramay H Microbiome-derived inosine modulates response to checkpoint inhibitor immunotherapy. Science. 2020;369:1481–9. 10.1126/science.abc3421 32792462

[111] Gao R Kong C Huang L Li H Qu X Liu Z Mucosa-associated microbiota signature in colorectal cancer. Eur J Clin Microbiol Infect Dis. 2017;36:2073–83. 10.1007/s10096-017-3026-4 28600626

[112] Koi M Okita Y Carethers JM. *Fusobacterium nucleatum* infection in colorectal cancer: linking lnflammation, DNA mismatch repair and genetic and epigenetic alterations. J Anus Rectum Colon. 2018;2:37–46. 10.23922/jarc.2017-055 30116794 PMC6090547

[113] Lee DW Han SW Kang JK Bae JM Kim HP Won JK Association between *Fusobacterium nucleatum*, pathway mutation, and patient prognosis in colorectal cancer. Ann Surg Oncol. 2018;25:3389–95. 10.1245/s10434-018-6681-5 30062471

[114] Yu M Kim JK Kim SY Cho SH Kim MJ Seomun G. Development and effects of simulation program for fall management. J Korean Acad Nurs Adm. 2017;23:548–57. 10.11111/jkana.2017.23.5.548

[115] Zhou Z Chen J Yao H Hu H. *Fusobacterium* and colorectal cancer. Front Oncol. 2018;8:371. 10.3389/fonc.2018.00371 30374420 PMC6196248

[116] Yang Y Weng W Peng J Hong L Yang L Toiyama Y *Fusobacterium nucleatum* increases proliferation of colorectal cancer cells and tumor development in mice by activating toll-like receptor 4 signaling to nuclear factor-κB, and up-regulating expression of microRNA-21. Gastroenterology. 2017;152:851–66.E24. 10.1053/j.gastro.2016.11.018 27876571 PMC5555435

[117] Wang HB Wang PY Wang X Wan YL Liu YC. Butyrate enhances intestinal epithelial barrier function via up-regulation of tight junction protein claudin-1 transcription. Dig Dis Sci. 2012;57:3126–35. 10.1007/s10620-012-2259-4 22684624

[118] Lam KC Vyshenska D Hu J Rodrigues RR Nilsen A Zielke RA Transkingdom network reveals bacterial players associated with cervical cancer gene expression program. PeerJ. 2018;6:e5590. 10.7717/peerj.5590 30294508 PMC6170155

[119] Liu Y Lin Z Lin Y Chen Y Peng XE He F *Streptococcus* and *prevotella* are associated with the prognosis of oesophageal squamous cell carcinoma. J Med Microbiol. 2018;67:1058–68. 10.1099/jmm.0.000754 29923815

[120] Nejman D Livyatan I Fuks G Gavert N Zwang Y Geller LT The human tumor microbiome is composed of tumor type–specific intracellular bacteria. Science. 2020;368:973–80. 10.1126/science.aay9189 32467386 PMC7757858

[121] Bertocchi A Carloni S Ravenda PS Bertalot G Spadoni I Lo Cascio A Gut vascular barrier impairment leads to intestinal bacteria dissemination and colorectal cancer metastasis to liver. Cancer Cell. 2021;39:708–24.E11. 10.1016/j.ccell.2021.03.004 33798472

[122] Łaniewski P Ilhan ZE Herbst-Kralovetz MM. The microbiome and gynaecological cancer development, prevention and therapy. Nat Rev Urol. 2020;17:232–50. 10.1038/s41585-020-0286-z 32071434 PMC9977514

[123] Del Castillo E Meier R Chung M Koestler DC Chen T Paster BJ The microbiomes of pancreatic and duodenum tissue overlap and are highly subject specific but differ between pancreatic cancer and noncancer subjects. Cancer Epidemiol Biomarkers Prev. 2019;28:370–83. 10.1158/1055-9965.EPI-18-0542 30373903 PMC6363867

[124] Bekliz M Colson P La Scola B. The expanding family of virophages. Viruses. 2016;8:317. 10.3390/v8110317 27886075 PMC5127031

[125] Yinda CK Vanhulle E Conceição-Neto N Beller L Deboutte W Shi C Gut virome analysis of cameroonians reveals high diversity of enteric viruses, including potential interspecies transmitted viruses. mSphere. 2019;4:e00585–18. 10.1128/mSphere.00585-18 30674646 PMC6344602

[126] Muresu N Sotgiu G Saderi L Sechi I Cossu A Marras V Distribution of HPV genotypes in patients with a diagnosis of anal cancer in an Italian region. Int J Environ Res Public Health. 2020;17:4516. 10.3390/ijerph17124516 32585996 PMC7345529

[127] Cantalupo PG Katz JP Pipas JM. Viral sequences in human cancer. Virology. 2018;513:208–16. 10.1016/j.virol.2017.10.017 29107929 PMC5828528

[128] Mollerup S Asplund M Friis-Nielsen J Kjartansdóttir KR Fridholm H Hansen TA High-throughput sequencing-based investigation of viruses in human cancers by multienrichment approach. J Infect Dis. 2019;220:1312–24. 10.1093/infdis/jiz318 31253993 PMC6743825

[129] Mesri EA Feitelson MA Munger K. Human viral oncogenesis: a cancer hallmarks analysis. Cell Host Microbe. 2014;15:266–82. 10.1016/j.chom.2014.02.011 24629334 PMC3992243

[130] Zhang LL Wei JY Wang L Huang SL Chen JL. Human T-cell lymphotropic virus type 1 and its oncogenesis. Acta Pharmacol Sin. 2017;38:1093–103. 10.1038/aps.2017.17 28392570 PMC5547553

[131] Marônek M Link R Monteleone G Gardlík R Stolfi C. Viruses in cancers of the digestive system: active contributors or idle bystanders? Int J Mol Sci. 2020;21:8133. 10.3390/ijms21218133 33143318 PMC7663754

[132] de Villiers EM Fauquet C Broker TR Bernard HU zur Hausen H. Classification of papillomaviruses. Virology. 2004;324:17–27. 10.1016/j.virol.2004.03.033 15183049

[133] Doorbar J Egawa N Griffin H Kranjec C Murakami I. Human papillomavirus molecular biology and disease association. Rev Med Virol. 2015;25:2–23. 10.1002/rmv.1822 25752814 PMC5024016

[134] Buitrago-Pérez A Garaulet G Vázquez-Carballo A Paramio JM García-Escudero R. Molecular signature of HPV-induced carcinogenesis: pRb, p53 and gene expression profiling. Curr Genomics. 2009;10:26–34. 10.2174/138920209787581235 19721808 PMC2699838

[135] Emlet C Ruffin M Lamendella R. Enteric virome and carcinogenesis in the gut. Dig Dis Sci. 2020;65:852–64. 10.1007/s10620-020-06126-4 32060814

[136] Chen W Li F Mead L White H Walker J Ingram DA Human papillomavirus causes an angiogenic switch in keratinocytes which is sufficient to alter endothelial cell behavior. Virology. 2007;367:168–74. 10.1016/j.virol.2007.05.030 17602722 PMC2043482

[137] Zwerschke W Mazurek S Massimi P Banks L Eigenbrodt E Jansen-Dürr P. Modulation of type M2 pyruvate kinase activity by the human papillomavirus type 16 E7 oncoprotein. Proc Natl Acad Sci U S A. 1999;96:1291–6. 10.1073/pnas.96.4.1291 9990017 PMC15456

[138] Spangle JM Munger K. The HPV16 E6 oncoprotein causes prolonged receptor protein tyrosine kinase signaling and enhances internalization of phosphorylated receptor species. PLoS Pathog. 2013;9:e1003237. 10.1371/journal.ppat.1003237 23516367 PMC3597533

[139] Chen HP Jiang JK Chan CH Teo WH Yang CY Chen YC Genetic polymorphisms of the human cytomegalovirus *UL144* gene in colorectal cancer and its association with clinical outcome. J Gen Virol. 2015;96:3613–23. 10.1099/jgv.0.000308 26450180

[140] Fiorina L Ricotti M Vanoli A Luinetti O Dallera E Riboni R Systematic analysis of human oncogenic viruses in colon cancer revealed EBV latency in lymphoid infiltrates. Infect Agent Cancer. 2014;9:18. 10.1186/1750-9378-9-18 24936208 PMC4058445

[141] Song LB Zhang X Zhang CQ Zhang Y Pan ZZ Liao WT Infection of Epstein-Barr virus in colorectal cancer in Chinese. Ai Zheng. 2006;25:1356–60. 17094901

[142] Awerkiew S Bollschweiler E Metzger R Schneider PM Hölscher AH Pfister H. Esophageal cancer in Germany is associated with Epstein-Barr-virus but not with papillomaviruses. Med Microbiol Immunol. 2003;192:137–40. 10.1007/s00430-002-0128-z 12920588

[143] Nakatsu G Zhou H Wu WKK Wong SH Coker OO Dai Z Alterations in enteric virome are associated with colorectal cancer and survival outcomes. Gastroenterology. 2018;155:529–41.E5. 10.1053/j.gastro.2018.04.018 29689266

[144] Johnson CH Dejea CM Edler D Hoang LT Santidrian AF Felding BH Metabolism links bacterial biofilms and colon carcinogenesis. Cell Metab. 2015;21:891–7. 10.1016/j.cmet.2015.04.011 25959674 PMC4456201

[145] Gao R Zhu Y Kong C Xia K Li H Zhu Y Alterations, interactions, and diagnostic potential of gut bacteria and viruses in colorectal cancer. Front Cell Infect Microbiol. 2021;11:657867. 10.3389/fcimb.2021.657867 34307189 PMC8294192

[146] Massimino L Lovisa S Antonio Lamparelli L Danese S Ungaro F. Gut eukaryotic virome in colorectal carcinogenesis: is that a trigger? Comput Struct Biotechnol J. 2020;19:16–28. 10.1016/j.csbj.2020.11.055 33363706 PMC7750180

[147] Marongiu L Landry JJM Rausch T Abba ML Delecluse S Delecluse HJ Metagenomic analysis of primary colorectal carcinomas and their metastases identifies potential microbial risk factors. Mol Oncol. 2021;15:3363–84. 10.1002/1878-0261.13070 34328665 PMC8637581

[148] Ackerman AL Underhill DM. The mycobiome of the human urinary tract: potential roles for fungi in urology. Ann Transl Med. 2017;5:31. 10.21037/atm.2016.12.69 28217696 PMC5300854

[149] Delecluse S Tsai MH Shumilov A Bencun M Arrow S Beshirova A Epstein-Barr virus induces expression of the LPAM-1 Integrin in B cells *in vitro* and *in vivo*. J Virol. 2019;93:e01618–18. 10.1128/JVI.01618-18 30541846 PMC6384065

[150] Ma W Mao Q Xia W Dong G Yu C Jiang F. Gut microbiota shapes the efficiency of cancer therapy. Front Microbiol. 2019;10:1050. 10.3389/fmicb.2019.01050 31293523 PMC6604670

[151] Samet A Sledzińska A Krawczyk B Hellmann A Nowicki S Kur J Leukemia and risk of recurrent *Escherichia coli* bacteremia: genotyping implicates *E. coli* translocation from the colon to the bloodstream. Eur J Clin Microbiol Infect Dis. 2013;32:1393–400. 10.1007/s10096-013-1886-9 23649557 PMC3824565

[152] Wolochow H Hildebrand GJ Lamanna C. Translocation of microorganisms across the intestinal wall of the rat: effect of microbial size and concentration. J Infect Dis. 1966;116:523–8. 10.1093/infdis/116.4.523 4959185

[153] Perez-Chanona E Trinchieri G. The role of microbiota in cancer therapy. Curr Opin Immunol. 2016;39:75–81. 10.1016/j.coi.2016.01.003 26820225 PMC4801762

[154] Erdman SE Poutahidis T. Gut microbiota modulate host immune cells in cancer development and growth. Free Radic Biol Med. 2017;105:28–34. 10.1016/j.freeradbiomed.2016.11.013 27840315 PMC5831246

[155] Viaud S Saccheri F Mignot G Yamazaki T Daillère R Hannani D The intestinal microbiota modulates the anticancer immune effects of cyclophosphamide. Science. 2013;342:971–6. 10.1126/science.1240537 24264990 PMC4048947

[156] Daillère R Vétizou M Waldschmitt N Yamazaki T Isnard C Poirier-Colame V *Enterococcus hirae* and *Barnesiella intestinihominis* facilitate cyclophosphamide-induced therapeutic immunomodulatory effects. Immunity. 2016;45:931–43. 10.1016/j.immuni.2016.09.009 27717798

[157] Kroemer G Galluzzi L Kepp O Zitvogel L. Immunogenic cell death in cancer therapy. Annu Rev Immunol. 2013;31:51–72. 10.1146/annurev-immunol-032712-100008 23157435

[158] Scott TA Quintaneiro LM Norvaisas P Lui PP Wilson MP Leung KY Host-microbe co-metabolism dictates cancer drug efficacy in *C. elegans*. Cell. 2017;169:442–56.E18. 10.1016/j.cell.2017.03.040 28431245 PMC5406385

[159] Kim DH. Gut microbiota-mediated drug-antibiotic interactions. Drug Metab Dispos. 2015;43:1581–9. 10.1124/dmd.115.063867 25926432

[160] Iida N Dzutsev A Stewart CA Smith L Bouladoux N Weingarten RA Commensal bacteria control cancer response to therapy by modulating the tumor microenvironment. Science. 2013;342:967–70. 10.1126/science.1240527 24264989 PMC6709532

[161] Klaassen CD Cui JY. Review: mechanisms of how the intestinal microbiota alters the effects of drugs and bile acids. Drug Metab Dispos. 2015;43:1505–21. 10.1124/dmd.115.065698 26261286 PMC4576672

[162] García-González AP Ritter AD Shrestha S Andersen EC Yilmaz LS Walhout AJM. Bacterial metabolism affects the *C. elegans* response to cancer chemotherapeutics. Cell. 2017;169:431–41.E8. 10.1016/j.cell.2017.03.046 28431244 PMC5484065

[163] Guthrie L Gupta S Daily J Kelly L. Human microbiome signatures of differential colorectal cancer drug metabolism. NPJ Biofilms Microbiomes. 2017;3:27. 10.1038/s41522-017-0034-1 29104759 PMC5665930

[164] Fijlstra M Ferdous M Koning AM Rings EH Harmsen HJ Tissing WJ. Substantial decreases in the number and diversity of microbiota during chemotherapy-induced gastrointestinal mucositis in a rat model. Support Care Cancer. 2015;23:1513–22. 10.1007/s00520-014-2487-6 25376667

[165] Montassier E Gastinne T Vangay P Al-Ghalith GA Bruley des Varannes S Massart S Chemotherapy-driven dysbiosis in the intestinal microbiome. Aliment Pharmacol Ther. 2015;42:515–28. 10.1111/apt.13302 26147207

[166] Yaguchi T Sumimoto H Kudo-Saito C Tsukamoto N Ueda R Iwata-Kajihara T The mechanisms of cancer immunoescape and development of overcoming strategies. Int J Hematol. 2011;93:294–300. 10.1007/s12185-011-0799-6 21374075

[167] Zhang Y Rajput A Jin N Wang J. Mechanisms of immunosuppression in colorectal cancer. Cancers (Basel). 2020;12:3850. 10.3390/cancers12123850 33419310 PMC7766388

[168] Lu Y Yuan X Wang M He Z Li H Wang J Gut microbiota influence immunotherapy responses: mechanisms and therapeutic strategies. J Hematol Oncol. 2022;15:47. 10.1186/s13045-022-01273-9 35488243 PMC9052532

[169] Dillman RO. Cancer immunotherapy. Cancer Biother Radiopharm. 2011;26:1–64. 10.1089/cbr.2010.0902 21355777

[170] Esfahani K Roudaia L Buhlaiga N Del Rincon SV Papneja N Miller WH Jr. A review of cancer immunotherapy: from the past, to the present, to the future. Curr Oncol. 2020;27:S87–97. 10.3747/co.27.5223 32368178 PMC7194005

[171] Rausch MP Hastings KT. Immune checkpoint inhibitors in the treatment of melanoma: from basic science to clinical application. In: Ward WH Farma JM, editors. Cutaneous melanoma: etiology and therapy. Exon Publications; 2017. pp. 121–42. 10.15586/codon.cutaneousmelanoma.2017.ch9 29461774

[172] Harrington KJ Andtbacka RH Collichio F Downey G Chen L Szabo Z Efficacy and safety of talimogene laherparepvec *versus* granulocyte-macrophage colony-stimulating factor in patients with stage IIIB/C and IVM1a melanoma: subanalysis of the phase III OPTiM trial. Onco Targets Ther. 2016;9:7081–93. 10.2147/OTT.S115245 27895500 PMC5119624

[173] Chen L Douglass J Kleinberg L Ye X Marciscano AE Forde PM Concurrent immune checkpoint inhibitors and stereotactic radiosurgery for brain metastases in non-small cell lung cancer, melanoma, and renal cell carcinoma. Int J Radiat Oncol Biol Phys. 2018;100:916–25. 10.1016/j.ijrobp.2017.11.041 29485071

[174] Kobayashi T Iwama S Yasuda Y Okada N Okuji T Ito M Pituitary dysfunction induced by immune checkpoint inhibitors is associated with better overall survival in both malignant melanoma and non-small cell lung carcinoma: a prospective study. J Immunother Cancer. 2020;8:e000779. 10.1136/jitc-2020-000779 32606047 PMC7328763

[175] Restifo NP Smyth MJ Snyder A. Acquired resistance to immunotherapy and future challenges. Nat Rev Cancer. 2016;16:121–6. 10.1038/nrc.2016.2 26822578 PMC6330026

[176] Corbaux P Maillet D Boespflug A Locatelli-Sanchez M Perier-Muzet M Duruisseaux M Older and younger patients treated with immune checkpoint inhibitors have similar outcomes in real-life setting. Eur J Cancer. 2019;121:192–201. 10.1016/j.ejca.2019.08.027 31590080

[177] Kabat AM Srinivasan N Maloy KJ. Modulation of immune development and function by intestinal microbiota. Trends Immunol. 2014;35:507–17. 10.1016/j.it.2014.07.010 25172617 PMC6485503

[178] Qiu Q Lin Y Ma Y Li X Liang J Chen Z Exploring the emerging role of the gut microbiota and tumor microenvironment in cancer immunotherapy. Front Immunol. 2021;11:612202. 10.3389/fimmu.2020.612202 33488618 PMC7817884

[179] Matson V Chervin CS Gajewski TF. Cancer and the microbiome-influence of the commensal microbiota on cancer, immune responses, and immunotherapy. Gastroenterology. 2021;160:600–13. 10.1053/j.gastro.2020.11.041 33253684 PMC8409239

[180] Della Vittoria Scarpati G Fusciello C Perri F Sabbatino F Ferrone S Carlomagno C Ipilimumab in the treatment of metastatic melanoma: management of adverse events. Onco Targets Ther. 2014;7:203–9. 10.2147/OTT.S57335 24570590 PMC3933725

[181] Chaput N Lepage P Coutzac C Soularue E Le Roux K Monot C Baseline gut microbiota predicts clinical response and colitis in metastatic melanoma patients treated with ipilimumab. Ann Oncol. 2019;30:2012. Erratum in: Ann Oncol. 2017;28:1368–79. 10.1093/annonc/mdz224 28368458

[182] Pitt JM Vétizou M Gomperts Boneca I Lepage P Chamaillard M Zitvogel L. Enhancing the clinical coverage and anticancer efficacy of immune checkpoint blockade through manipulation of the gut microbiota. Oncoimmunology. 2016;6:e1132137. 10.1080/2162402X.2015.1132137 28197360 PMC5283646

[183] Kim E Ahn H Park H. A review on the role of gut microbiota in immune checkpoint blockade therapy for cancer. Mamm Genome. 2021;32:223–31. 10.1007/s00335-021-09867-3 33783613 PMC8295158

[184] Miller PL Carson TL. Mechanisms and microbial influences on CTLA-4 and PD-1-based immunotherapy in the treatment of cancer: a narrative review. Gut Pathog. 2020;12:43. 10.1186/s13099-020-00381-6 32944086 PMC7488430

[185] Bianco A Perrotta F Barra G Malapelle U Rocco D De Palma R. Prognostic factors and biomarkers of responses to immune checkpoint inhibitors in lung cancer. Int J Mol Sci. 2019;20:4931. 10.3390/ijms20194931 31590386 PMC6801651

[186] Routy B Le Chatelier E Derosa L Duong CPM Alou MT Daillère R Gut microbiome influences efficacy of PD-1-based immunotherapy against epithelial tumors. Science. 2018;359:91–7. 10.1126/science.aan3706 29097494

[187] Elkrief A Derosa L Zitvogel L Kroemer G Routy B. The intimate relationship between gut microbiota and cancer immunotherapy. Gut Microbes. 2019;10:424–8. 10.1080/19490976.2018.1527167 30339501 PMC6546322

[188] Shaikh FY Gills JJ Sears CL. Impact of the microbiome on checkpoint inhibitor treatment in patients with non-small cell lung cancer and melanoma. EBioMedicine. 2019;48:642–7. 10.1016/j.ebiom.2019.08.076 31597596 PMC6838599

[189] Chen H Cong X Wu C Wu X Wang J Mao K Intratumoral delivery of CCL25 enhances immunotherapy against triple-negative breast cancer by recruiting CCR9^+^ T cells. Sci Adv. 2020;6:eaax4690. 10.1126/sciadv.aax4690 32064335 PMC6989134

[190] Ribas A Dummer R Puzanov I VanderWalde A Andtbacka RHI Michielin O Oncolytic virotherapy promotes intratumoral T cell infiltration and improves anti-PD-1 iImmunotherapy. Cell. 2017;170:1109–19.E10. Erratum in: Cell. 2018;174:1031–2. 10.1016/j.cell.2017.08.027 30096300

[191] Weiss SA Sznol M. Resistance mechanisms to checkpoint inhibitors. Curr Opin Immunol. 2021;69:47–55. 10.1016/j.coi.2021.02.001 33676271

[192] Panebianco C Andriulli A Pazienza V. Pharmacomicrobiomics: exploiting the drug-microbiota interactions in anticancer therapies. Microbiome. 2018;6:92. 10.1186/s40168-018-0483-7 29789015 PMC5964925

[193] Nagano T Otoshi T Hazama D Kiriu T Umezawa K Katsurada N Novel cancer therapy targeting microbiome. Onco Targets Ther. 2019;12:3619–24. 10.2147/OTT.S207546 31190864 PMC6526180

[194] Samaan MA Pavlidis P Papa S Powell N Irving PM. Gastrointestinal toxicity of immune checkpoint inhibitors: from mechanisms to management. Nat Rev Gastroenterol Hepatol. 2018;15:222–34. 10.1038/nrgastro.2018.14 29512649

[195] Lawrie TA Green JT Beresford M Wedlake L Burden S Davidson SE Interventions to reduce acute and late adverse gastrointestinal effects of pelvic radiotherapy for primary pelvic cancers. Cochrane Database Syst Rev. 2018;1:CD012529. 10.1002/14651858.CD012529.pub2 29360138 PMC6491191

[196] Bajic JE Johnston IN Howarth GS Hutchinson MR. From the bottom-up: chemotherapy and gut-brain axis dysregulation. Front Behav Neurosci. 2018;12:104. 10.3389/fnbeh.2018.00104 29872383 PMC5972222

[197] Zitvogel L Ma Y Raoult D Kroemer G Gajewski TF. The microbiome in cancer immunotherapy: diagnostic tools and therapeutic strategies. Science. 2018;359:1366–70. 10.1126/science.aar6918 29567708

[198] Redman MG Ward EJ Phillips RS. The efficacy and safety of probiotics in people with cancer: a systematic review. Ann Oncol. 2014;25:1919–29. 10.1093/annonc/mdu106 24618152

[199] Mego M Holec V Drgona L Hainova K Ciernikova S Zajac V. Probiotic bacteria in cancer patients undergoing chemotherapy and radiation therapy. Complement Ther Med. 2013;21:712–23. 10.1016/j.ctim.2013.08.018 24280481

[200] Gianotti L Morelli L Galbiati F Rocchetti S Coppola S Beneduce A A randomized double-blind trial on perioperative administration of probiotics in colorectal cancer patients. World J Gastroenterol. 2010;16:167–75. 10.3748/wjg.v16.i2.167 20066735 PMC2806554

[201] Jiang C Wang H Xia C Dong Q Chen E Qiu Y A randomized, double-blind, placebo-controlled trial of probiotics to reduce the severity of oral mucositis induced by chemoradiotherapy for patients with nasopharyngeal carcinoma. Cancer. 2019;125:1081–90. 10.1002/cncr.31907 30521105

[202] Schroeder BO Birchenough GMH Ståhlman M Arike L Johansson MEV Hansson GC Bifidobacteria or fiber protects against diet-induced microbiota-mediated colonic mucus deterioration. Cell Host Microbe. 2018;23:27–40.E7. 10.1016/j.chom.2017.11.004 29276171 PMC5764785

[203] Wang LS Mo YY Huang YW Echeveste CE Wang HT Chen J Effects of dietary interventions on gut microbiota in humans and the possible impacts of foods on patients’ responses to cancer immunotherapy. eFood. 2020;1:279–87. 10.2991/efood.k.200824.002 34308386 PMC8301224

[204] Brodmann T Endo A Gueimonde M Vinderola G Kneifel W de Vos WM Safety of novel microbes for human consumption: practical examples of assessment in the European Union. Front Microbiol. 2017;8:1725. 10.3389/fmicb.2017.01725 28955311 PMC5601064

[205] Grigorescu I Dumitrascu DL. Implication of gut microbiota in diabetes mellitus and obesity. Acta Endocrinol (Buchar). 2016;12:206–14. 10.4183/aeb.2016.206 31149088 PMC6535288

[206] Napolitano M Covasa M. Microbiota transplant in the treatment of obesity and diabetes: current and future perspectives. Front Microbiol. 2020;11:590370. 10.3389/fmicb.2020.590370 33304339 PMC7693552

[207] Gopalakrishnan V Spencer CN Nezi L Reuben A Andrews MC Karpinets TV Gut microbiome modulates response to anti–PD-1 immunotherapy in melanoma patients. Science. 2018;359:97–103. 10.1126/science.aan4236 29097493 PMC5827966

[208] Matson V Fessler J Bao R Chongsuwat T Zha Y Alegre ML The commensal microbiome is associated with anti–PD-1 efficacy in metastatic melanoma patients. Science. 2018;359:104–8. 10.1126/science.aao3290 29302014 PMC6707353

[209] Bao HD Pang MD Olaniran A Zhang XH Zhang H Zhou Y Alterations in the diversity and composition of mice gut microbiota by lytic or temperate gut phage treatment. Appl Microbiol Biotechnol. 2018;102:10219–30. 10.1007/s00253-018-9378-6 30302521

[210] Rasmussen TS Mentzel CMJ Kot W Castro-Mejía JL Zuffa S Swann JR Faecal virome transplantation decreases symptoms of type 2 diabetes and obesity in a murine model. Gut. 2020;69:2122–30. 10.1136/gutjnl-2019-320005 32165408

[211] Draper LA Ryan FJ Smith MK Jalanka J Mattila E Arkkila PA Long-term colonisation with donor bacteriophages following successful faecal microbial transplantation. Microbiome. 2018;6:220. 10.1186/s40168-018-0598-x 30526683 PMC6288847

[212] Hsu BB Gibson TE Yeliseyev V Liu Q Lyon L Bry L Dynamic modulation of the gut microbiota and metabolome by bacteriophages in a mouse model. Cell Host Microbe. 2019;25:803–14.E5. 10.1016/j.chom.2019.05.001 31175044 PMC6579560

